# GSDMEa-mediated pyroptosis is bi-directionally regulated by caspase and required for effective bacterial clearance in teleost

**DOI:** 10.1038/s41419-022-04896-5

**Published:** 2022-05-24

**Authors:** Hang Xu, Shuai Jiang, Chao Yu, Zihao Yuan, Li Sun

**Affiliations:** 1 CAS and Shandong Province Key Laboratory of Experimental Marine Biology, Institute of Oceanology; CAS Center for Ocean Mega-Science, Chinese Academy of Sciences, Qingdao, China; 2 Laboratory for Marine Biology and Biotechnology, Pilot National Laboratory for Marine Science and Technology, Qingdao, China; 3 University of Chinese Academy of Sciences, Beijing, China

**Keywords:** Cell death and immune response, Cell death

## Abstract

Gasdermin (GSDM) is a family of pore-forming proteins that, after cleavage by caspase (CASP), induce a type of programmed necrotic cell death called pyroptosis. Gasdermin E (GSDME) is the only pyroptosis-inducing member of the GSDM family existing in teleost. To date, the regulation and function of teleost GSDME in response to bacterial infection remain elusive. In this study, we observed activation of GSDME, as well as multiple CASPs, in turbot *Scophthalmus maximus* during the infection of the bacterial pathogen *Vibrio harveyi*. Turbot has two GSDME orthologs named SmGSDMEa and SmGSDMEb. We found that SmGSDMEa was specifically cleaved by turbot CASP (SmCASP) 3/7 and SmCASP6, which produced two different N-terminal (NT) fragments. Only the NT fragment produced by SmCASP3/7 cleavage was able to induce pyroptosis. Ectopically expressed SmCASP3/7 activated SmGSDMEa, resulting in pyroptotic cell death. In contrast, SmCASP6 inactivated SmGSDMEa by destructive cleavage of the NT domain, thus nullifying the activation effect of SmCASP3/7. Unlike SmGSDMEa, SmGSDMEb was cleaved by SmCASP8 and unable to induce cell death. *V. harveyi* infection dramatically promoted the production and activation of SmGSDMEa, but not SmGSDMEb, and caused pyroptosis in turbot. Interference with SmCASP3/7 activity significantly enhanced the invasiveness and lethality of *V. harveyi* in a turbot infection model. Together, these results revealed a previously unrecognized bi-directional regulation mode of GSDME-mediated pyroptosis, and a functional difference between teleost GSDMEa and GSDMEb in the immune defense against bacterial infection.

## Introduction

Pyroptosis is a form of programmed necrotic cell death that provokes robust immune response and releases pro-inflammatory cytokines [1]. Gasdermin (GSDM) is the direct executioner of pyroptosis [2–4]. In human, there exist six GSDM family members named GSDMA to F. Except for GSDMF, all GSDMs are composed of a conserved N-terminal (NT) domain and a C-terminal (CT) domain [5]. Cleavage of GSDM at the inter-domain linker region liberates the pore-forming NT domain, which subsequently translocates onto the cytoplasmic membrane and, after oligomerization, forms channels to trigger rapid osmotic swelling and finally cell lysis [6, 7]. In mammals, GSDME is cleaved specifically by caspase (CASP) 3 at the inter-domain linker region, and the released NT domain induces cell death switch from apoptosis to pyroptosis [8, 9]. GSDME-mediated pyroptosis has an intimate relationship with anti-microbial immunity. For example, vesicular stomatitis virus infection triggered CASP3-mediated GSDME cleavage in bone marrow-derived macrophages in mice, which switched cell death from apoptosis to pyroptosis [9]; H7N9 influenza virus infection elicited GSDME-mediated pyroptosis in alveolar epithelial cells, which led to persistent release of proinflammatory cytokines that formed lethal cytokine storm [10].

Different from mammals that have a panel of GSDM family members, teleost have only GSDME and GSDMF, and GSDME was proved to possess conserved pyroptosis-inducing capacity as its mammalian homologs [11]. To date, functional studies of GSDME have only been reported in three teleost species, i.e., zebrafish (*Danio rerio*), tongue sole (*Cynoglossus semilaevis*), and turbot (*Scophthalmus maximus*). In tongue sole and zebrafish, CASP1 and caspy2 (CASP4/5 functional homolog) cleaved GSDME and induced pyroptosis in response to lipopolysaccharide (LPS) stimulation or bacterial infection [12–14]. A different GSDME ortholog in zebrafish was cleaved by CASP3, and induced apoptosis-to-pyroptosis switch [8]. In turbot, a caspase that recognized a mammalian CASP5 substrate homolog (WEHD) was reported to be involved in GSDME-executed pyroptosis when ectopically expressed in a mammalian cell line [15].

The *Vibrio* genus has long been known to be the causative agent of a serious disease called vibriosis in both vertebrate and invertebrate [16, 17]. In mammals, especially in human, the pathogenesis of *Vibrio* sp. has attracted increasing attention [18]. Of the pathogenic members of this genus, *V. cholerae* induced CASP-independent necrosis in THP-1 monocytes [19]; *V. parahaemolyticus* infection induced autophagy in Hela cells [20]; *Vibrio vulnificus* activated apoptosis and necrosis in human intestinal epithelial cells [21]. *Vibrio*-mediated waterborne diseases were also reported to be involved in massive death of marine animals [22, 23]. *Vibrio harveyi* is one of the most widespread marine pathogens with a wide range of animal hosts [24]. In corals, *V. harveyi* induced severe necrotic disruption of coral tissues, and was considered to be the cause of the white syndrome in tropical stony corals [25]. In mollusk, increased abundance of *V. harveyi* in the host microbiome led to dramatic rise of mortality rate in the Pacific oyster *Crassostrea gigas* [26]. In fish, *V. harveyi* is a serious pathogen to a number of cultured marine fish, including turbot (*Scophthalmus maximus*) and Japanese flounder (*Paralichthys olivaceus*) [27]. *V. harveyi* was reported to be able to activate CASP3 and induce apoptosis [28, 29]. However, the molecular mechanism underlying *V. harveyi*-induced necrotic cell death remains unclear.

In the present study, by using a turbot infection model, we observed necrotic cell death and CASP activation caused by *V. harveyi*. These observations promoted us to investigate the involvement of GSDME-mediated pyroptosis in *V. harveyi*-induced necrosis. For this purpose, we examined the activation mechanism and function of turbot GSDME, which exists in two different forms named SmGSDMa and SmGSDMb. We found that it was SmGSDMa, not SmGSDMb, that played a vital role in bacterial infection, and that SmGSDMa and SmGSDMb were activated/cleaved via mechanisms different from that of previous understanding. Furthermore, our results revealed a bi-directional regulation of SmGSDMa-mediated pyroptosis by different CASPs. These results provide new insights into the activation mechanism and anti-bacterial function of GSDME in teleost.

## Results

### *V. harveyi* induces necrotic cell death and causes significant changes in GSDME expression and caspase activity in turbot

**Fig. 1:**
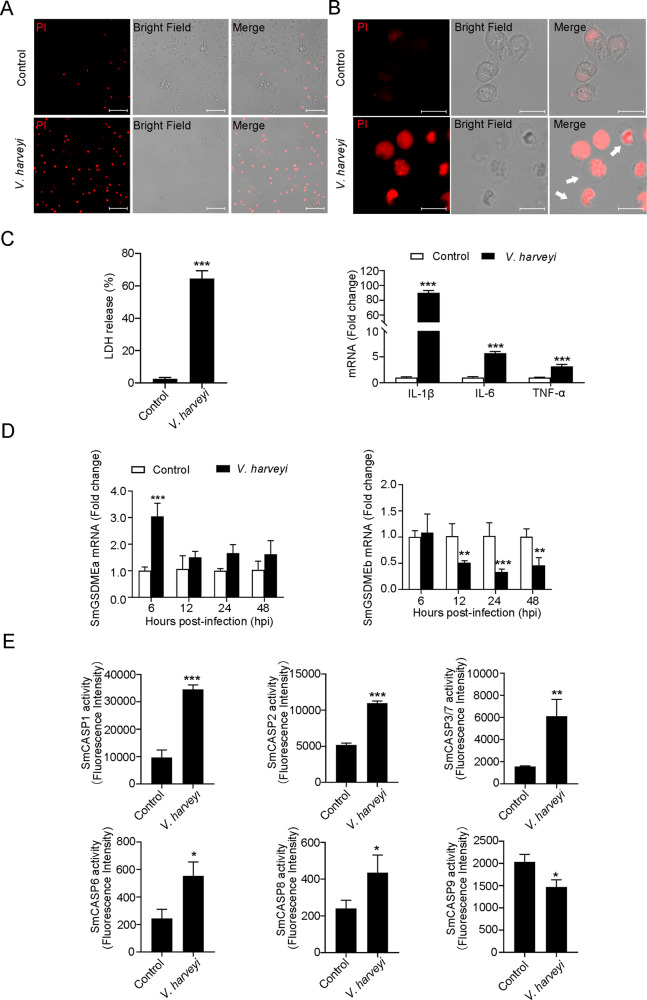
*Vibrio harveyi* infection induces necrotic cell death and alters SmGSDME expression and SmCASP activity. **A**, **B** Macrophages from *V. harveyi*-infected turbot were observed for PI uptake and morphological features of cell death. White arrows indicate cell death with swelling. Scale bars, 40 (**A**) and 10 (**B**) μm. **C** Turbot macrophages were infected with *V. harveyi* for 6 h and then measured for LDH release (left) and proinflammatory cytokine expression (right). **D** Turbot were infected with or without (control) *V. harveyi*, and SmGSDMEa and SmGSDMEb expression in kidney was determined by qRT-PCR at various time points. **E** Macrophages from *V. harveyi*-infected turbot were examined for SmCASPs activities. In all bar graphs, data represent the means ± SD, *n* = 3. ***P < 0.001; **P < 0.01; *P < 0.05.

In a live animal infection study, we found that when turbot were infected with the bacterial pathogen *V. harveyi*, marked cell death was observed in macrophages. The cells were highly susceptible to PI staining and became swollen up (Fig. 1A, B), suggesting necrosis. This was supported by the fact that *V. harveyi*-infected macrophages released massive amount of lactate dehydrogenase (LDH) and exhibited up-regulated expression of proinflammatory cytokines (Fig. 1C). Since GSDME-mediated pyroptosis plays an important role in necrosis [12–14], we examined the involvement of GSDME in *V. harveyi* infection. We found that the expressions of the two GSDME orthologs, which were named SmGSDMEa and SmGSDMEb based on the phylogenetic analysis of fish GSDMEa/b collected from the NCBI Orthologs (Fig. S1), occurred in eight turbot tissues, in particular immune related tissues (Fig. S2). During *V. harveyi* infection, SmGSDMEa and SmGSDMEb expressions were significantly upregulated and downregulated, respectively, in a time-dependent manner (Fig. 1D). Since caspase (CASP) cleavage is reported to be essential for GSDM activation, we also examined the effect of bacterial infection on the activity of turbot caspases (SmCASPs). We found that in the macrophages of *V. harveyi*-infected turbot, the activities of SmCASP1, 2, 3/7, 6, and 8 significantly increased, while the activity of SmCASP9 significantly decreased (Fig. 1E). Collectively, these results indicated that *V. harveyi* infection induced necrotic death combined with caspase activation and SmGSDME expression, which suggested the involvement of pyroptosis.

### SmGSDMEa is a substrate of CASP3, 6, and 7

**Fig. 2:**
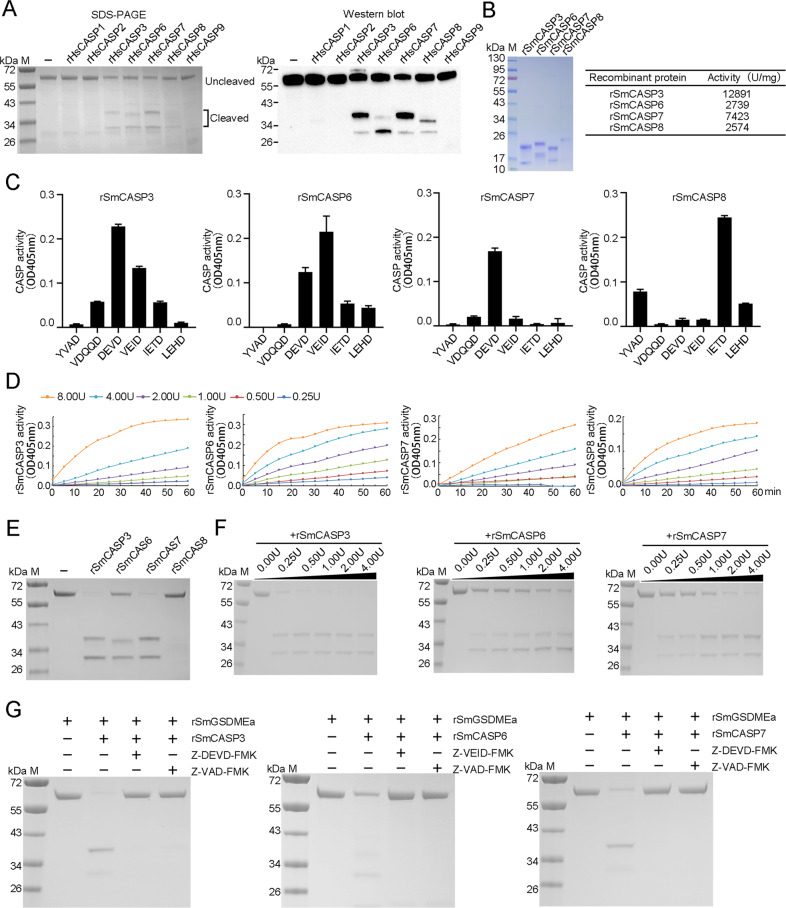
rSmGSDMEa is cleaved by CASP3/6/7. **A** rSmGSDMEa was treated with rHsCASP1, 2, 3, 6, 7, 8 or 9 and then analyzed by SDS-PAGE (left panel). The cleaved fragments were immunoblotted with anti-His tag antibody (right panel). **B** SDS-PAGE analysis (left) and activity (right) of rSmCASP3, 6, 7, and 8. **C** The proteolytic specificities of rSmCASP3, 6, 7 and 8 were determined by treatment with different colorimetric substrates and measuring the released ρNA. The data are expressed as the means ± SD, *n* = 3. **D** rSmCASP3, 6, 7, 8 at different doses (0.25 to 8 U) were incubated with their respective colorimetric substrates, and the released ρNA was measured. **E** rSmGSDMEa was treated with rSmCASP3, 6, 7, or 8, and then analyzed by SDS-PAGE. **F** rSmGSDMEa was treated with different units of rSmCASP3, 6 or 7, and then subjected to SDS-PAGE. **G** The cleavage of rSmGSDMEa by rSmCASP3, 6, or 7 were determined in the absence or presence of CASP inhibitors Z-VAD-FMK, Z-DEVD-FMK or Z-VEID-FMK.

The above observation, i.e., *V. harveyi* infection activated a cohort of CASPs, promoted us to examine whether these CASPs could cleave and activate SmGSDME. To facilitate this process, we first determined the susceptibility of recombinant SmGSDMEa (rSmGSDMEa) to a panel of recombinant human CASP (rHsCASP) that are commercially available. The results showed that rSmGSDMEa was cleaved by rHsCASP 3, 6, 7 and 8, but not by rHsCASP1, 2, or 9 (Fig. 2A). The cleavage profiles produced by rHsCASP3 and rHsCASP7 were identical, which differed from that produced by rHsCASP6 or 8. To examine the cleavage of rSmGSDEa by turbot CASPs, recombinant turbot CASP (rSmCASP) 3, 6, 7, and 8 were obtained (Fig. 2B). rSmCASP3, 6, 7, and 8 cleaved preferably, in a dose-dependent manner, the specific tetrapeptide substrates of their human counterparts (Fig. 2C, D). rSmCASP3/6/7 cleaved rSmGSDMEa into the NT and CT fragments similar to that cleaved by rHsCASP3/6/7, whereas rSmCASP8 failed to cleave rSmGSDMEa (Fig. 2E). The cleavage of rSmGSDMEa by rSmCASP3/6/7 was dependent on the dose of the CASPs (Fig. 2F). The presence of the CASP3 inhibitor (Z-DEVD-FMK) and the pan-CASP inhibitor (Z-VAD-FMK) completely blocked rSmCASP3/7 cleavage (Fig. 2G). Similarly, the presence of the CASP6 inhibitor (Z-VEID-FMK) and pan-CASP inhibitor effectively blocked rSmCASP6 cleavage (Fig. 2G). These results indicated that SmGSDMEa was a substrate of rSmCASP3/6/7, but not a substrate of rSmCASP8.

### SmCASP3/7 and SmCASP6 cleave SmGSDMEa at two different sites and produce two different NT fragments

**Fig. 3:**
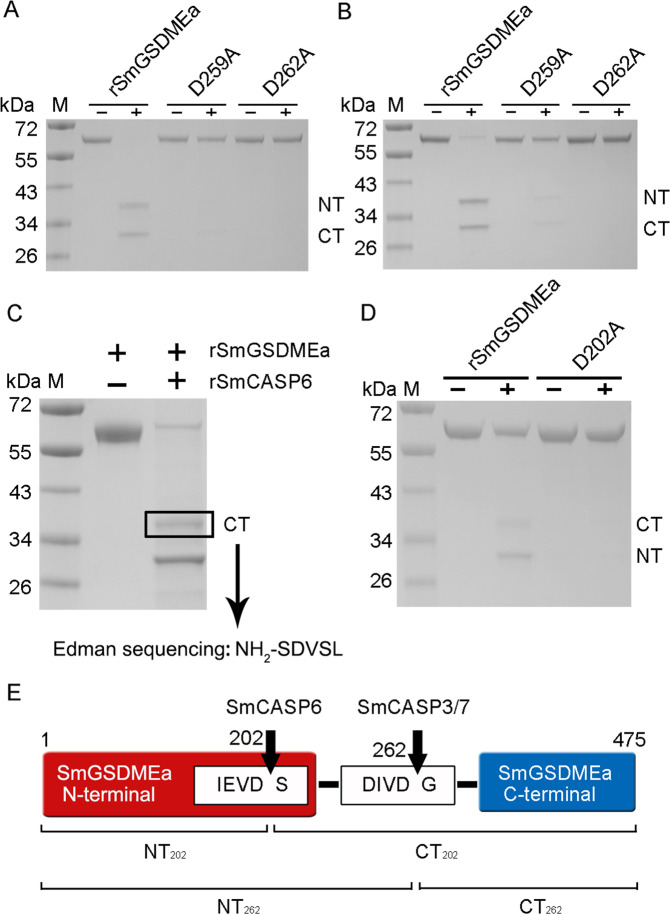
rSmCASP3/7 and rSmCASP6 cleave rSmGSDMEa at two different sites. **A**, **B** rSmGSDMEa and its mutants (D259A and D262A) were treated with rSmCASP3 or rSmCASP7. The products were analyzed by SDS-PAGE. **C** rSmGSDMEa was cleaved by rSmCASP6, and the resulting fragment was excised and subjected to Edman sequencing. **D** rSmGSDMEa and its mutant D202A were treated with rSmCASP6 and then subjected to SDS-PAGE. **E** A schematic presentation of the SmCASP3/6/7 cleavage sites in SmGSDMEa. For all panels, NT, N-terminal fragment; CT, C-terminal fragment.

Since, as shown above (Fig. 2C, D), rSmCASP3/7 preferably cleaved the DxxD motif, we analyzed the sequence of SmGSDMEa and found a _259_DIVD_262_ motif in the inter-domain linker region. We therefore examined whether this site was the target of rSmCASP3/7. For this purpose, two rSmGSDMEa mutants with Ala substitution at D259 and D262 (D259A and D262A) were created. Both mutants were completely resistant to rSmCASP3/7 cleavage (Fig. 3A, B), implying that _259_DIVD_262_ was indeed the cleavage site of rSmCASP3/7. To identify the cleavage site of rSmCASP6, four residues (D204, D227, D245 and D255) in the vicinity of the linker region of SmGSDMEa, were individually mutated to Ala. However, all mutants were unaffected in their cleavability by rSmCASP6 (data not shown). We then resorted to Edman sequencing to analyze the two rSmGSDMEa fragments generated by rSmCASP6 cleavage. The result showed that the larger fragment was the CT fragment, which bears _203_SDVSL_207_ at its N-terminus (Fig. 3C). Thus, the cleavage site should be between D^202^ and S^203^. Consistently, the mutant SmGSDMEa with D202A substitution became resistant to rSmCASP6 cleavage (Fig. 3D). Together, these results demonstrated that SmGSDMEa was cleaved by SmCASP3/7 and SmCASP6 at two distinct sites, i.e., _259_DIVD_262_ and _199_IEVD_202_, respectively (Fig. 3E). For convenience, the NT and CT fragments produced by rSmCASP3/7 cleavage at D262 were named SmGSDMEa-NT_262_ and SmGSDMEa-CT_262_, respectively. Likewise, the NT and CT fragments produced by rSmCASP6 cleavage at D202 were named SmGSDMEa-NT_202_ and SmGSDMEa-CT_202_, respectively.

### SmGSDMEa-NT_262_, but not -NT_202,_ is a pyroptosis inducer

**Fig. 4:**
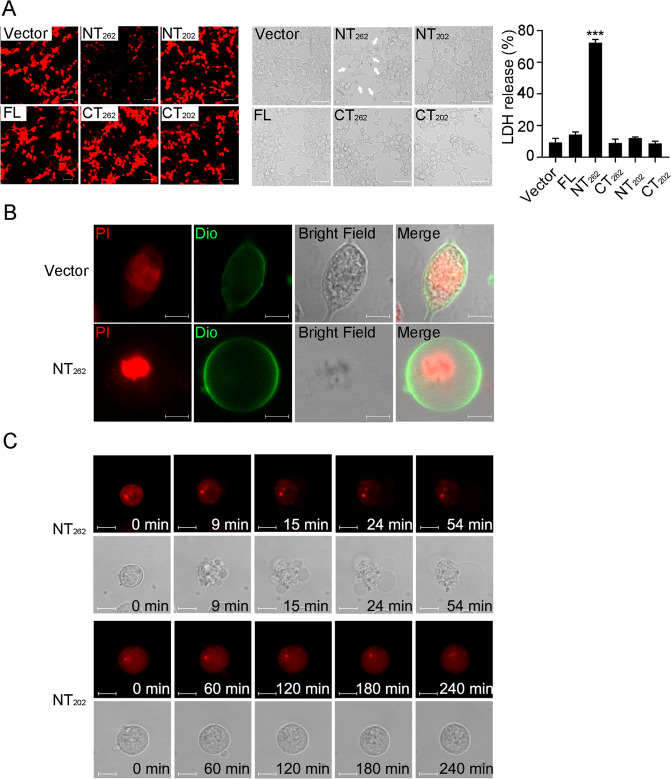
SmGSDMEa-NT_262_, but not SmGSDMEa-NT_202_, induces pyroptosis_._ **A** HEK293T cells were transfected with the backbone vector or the vector encoding mCherry-tagged SmGSDMEa-FL, -NT_262_, -CT_262_, -NT_202_, and -CT_202_ for 24 h. The cells were observed for the expression of the SmGSDMEa variants (left), morphological change (middle), and LDH release (right). Scale bar, 50 μm. Values are shown as means ± SD, *n* = 3. ***P < 0.001. **B** Confocal microscopy of HKE293T cells transfected with the backbone vector or the vector expressing SmGSDMEa-NT_262_. Cell nucleus and membrane were stained with PI and DiO, respectively. Scale bar, 10 μm. **C** HEK293T cells were transfected with mCherry-tagged SmGSDMEa-NT_262_ (upper panel) and SmGSDMEa-NT_202_ (lower panel), and time-lapse images were taken. Scale bar, 10 μm.

To examine whether SmGSDMEa-NT_262_ and SmGSDMEa-NT_202_ possess pyroptosis-inducing activity, mCherry-tagged SmGSDMEa-FL (full length SmGSDMEa), SmGSDMEa-NT (the NT fragment of SmGSDMEa), and SmGSDMEa-CT (the CT fragment of SmGSDMEa) were each expressed in HEK293T cells. Microscopy revealed that SmGSDMEa-FL, -CT_262_, -NT_202_, and -CT_202_ were expressed abundantly in the cells, whereas SmGSDMEa-NT_262_ expression was exceedingly low (Fig. 4A). Cells expressing SmGSDMEa-FL, -CT_262_, -NT_202_, and -CT_202_ exhibited normal morphology and barely released LDH, whereas cells expressing SmGSDMEa-NT_262_ released a large amount of LDH (Fig. 4A). SmGSDME-NT_262_ expression triggered typical pyroptotic cell death marked by osmotic swelling with the nucleus remaining intact (Fig. 4B). During this process, SmGSDMEa-NT_262_ induced, in a time-dependent manner, rapid membrane protrusion and bubbling that eventually led to membrane rupture and cell death (Fig. 4C). In contrast, SmGSDMEa-NT_202_ failed to trigger cell death (Fig. 4C). These results indicated that SmGSDMEa-NT_262_, not SmGSDMEa-NT_202_, was the executor of pyroptosis.

### Ectopically expressed SmCASP3/7 activate SmGSDMEa and cause pyroptotic cell death

**Fig. 5:**
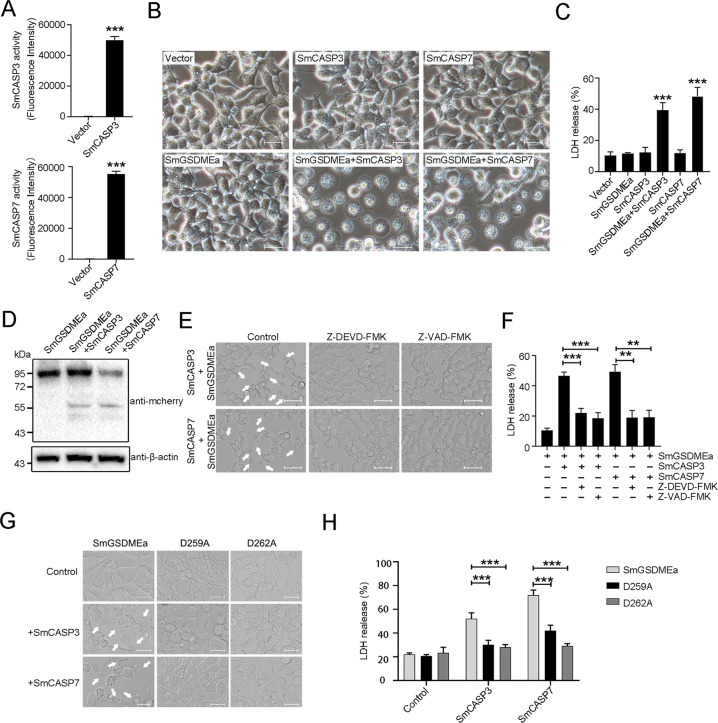
Ectopically expressed SmCASP3/7 cleave SmGSDMEa and induce pyroptosis. **A** The enzymatic activity of SmCASP3/7 in SmCASP3/7-transfected HKE293T cells was assessed. **B** HKE293T cells transfected alone with the backbone vector or the vector expressing SmGSDMEa, SmCASP3, and SmCASP7, or co-transfected with the vectors expressing SmGSDMEa plus SmCASP3 or SmGSDMEa plus SmCASP7. At 24 h post-transfection, the bright-field images were taken. Scale bar, 20 μm. **C** LDH released from the above transfected cells was measured. **D** The cleavage of SmGSDMEa in the above transfected cells was determined by immunoblot with anti-mCherry antibody. **E**, **F** HKE293T cells co-expressing SmGSDMEa and SmCASP3/7 in the presence or absence (control) of Z-VAD-FMK and Z-DEVD-FMK were subjected to microscopy and LDH measurement. White arrows indicate pyroptotic cells. Scale bar, 20 μm. **G**, **H** HEK293T cells co-transfected with the vectors expressing SmCASP3/7 plus SmGSDMEa or its mutants (D259A and D262A) were subjected to microscopy and LDH measurement. White arrows indicate pyroptotic cells. Scale bar, 20 μm. For panels **A**, **C**, **F**, and **H**, values are shown as means ± SD, *n* = 3. ***P < 0.001; **P < 0.01.

We next examined whether SmCASP3/6/7 could cleave and activate SmGSDMEa in a cellular system. For this purpose, SmCASP3/7 as well as SmGSDMEa were overexpressed in HEK293T cells (Fig. 5A, B). SmCASP3, SmCASP7, and SmGSDMEa expression alone had no apparent effect on cellular morphology and viability. However, co-expression of SmCASP3/7 and SmGSDMEa induced pyroptosis and massive release of LDH (Fig. 5B, C). Immunoblot detected the cleaved SmGSDMEa-CT fragment in co-transfected cells (Fig. 5D), implying activation of SmGSDMEa by SmCASP3/7. Consistently, the presence of the CASP3 inhibitor and pan-CASP inhibitor significantly inhibited pyroptosis and LDH release from the cells co-transfected with SmCASP3/7 and SmGSDMEa (Fig. 5E, F). The essentialness of SmCASP3/7 cleavage was further demonstrated by the observation that when the cells were co-transfected with SmCASP3/7 plus SmGSDMEa-D259A or SmGSDMEa-D262A, both pyroptosis and LDH release significantly decreased (Fig. 5G, H). These results indicated that SmCASP3/7 cleavage activated SmGSMDEa and enabled the latter to execute pyroptosis.

### SmCASP6 inactivates SmGSDMEa by destructive cleavage of the NT region

**Fig. 6:**
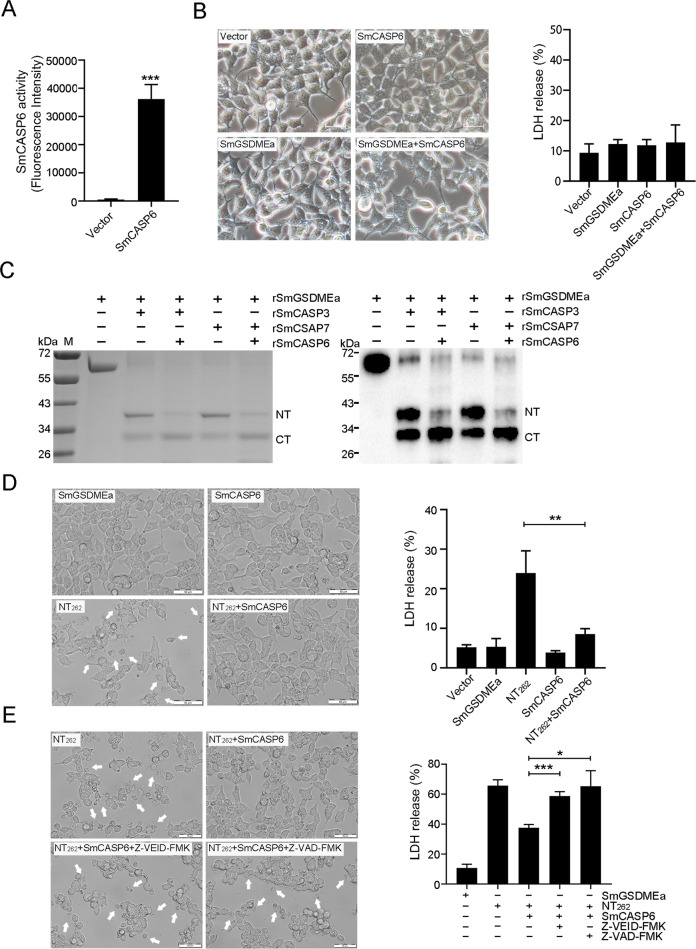
SmCASP6 inhibits SmGSDMEa activation. **A** The enzymatic activity of SmCASP6 in SmCASP6-transfected HKE293T cells was assessed. **B** Bright-field images of HKE293T cells transfected with the backbone vector or the vector expressing SmGSDMEa, SmCASP6, or SmGSDMEa plus SmCASP6 (left panel). Scale bar, 20 μm. LDH released from the transfected cells is shown on the right. **C** rSmGSDMEa was pre-incubated with rSmCASP3 or 7 and then treated with rSmCASP6 for 3 h. The products were subjected to SDS-PAGE (left) and immunoblot with anti-His tag antibody (right). **D** Bright-field images of HKE293T cells transfected with the backbone vector or the vector expressing SmGSDMEa, SmGSDMEa-NT_262_, SmCASP6, or SmGSDMEa-NT_262_ plus SmCASP6 (left). White arrows indicate pyroptotic cells. Scale bars, 50 μm. LDH released from the transfected cells is shown on the right. **E** HKE293T cells expressing SmGSDMEa-NT_262_ or SmGSDMEa-NT_262_ plus SmCASP6 in the presence or absence of Z-VAD-FMK and Z-VEID-FMK were subjected to microscopy (left) or LDH measurement (right). White arrows indicate pyroptotic cells. Scale bar, 50 μm. For panels **A**, **B**, **D**, and **E**, data are shown as means ± SD, *n* = 3. ***P < 0.001; **P < 0.01; *P < 0.05.

Since SmGSDMEa was also a substrate of SmCASP6, we examined whether SmCASP6 cleavage could activate SmGSDMEa at the cellular level. For this purpose, SmCASP6 and SmGSDMEa were expressed separately or together in HEK293T cells (Fig. 6A, B). Co-expression of SmCASP6 and SmGSDMEa led to no cell death or LDH release (Fig. 6B, C), which was consistent with the above observation that rSmGSDMEa-NT_202_ resulting from rSmCASP6 cleavage was incapable of inducing pyroptosis. Since SmGSDMEa-NT_262_ contains the recognition site of SmCASP6, we examined whether SmGSDMEa-NT_262_ could be digested by SmCASP6. We found that, indeed, the rSmGSDMEa-NT_262_ generated by both rSmCASP3 and 7 cleavages were further cleaved by rSmCASP6 (Fig. 6C). In line with this observation, co-expression of SmCASP6 with SmGSDMEa-NT_262_ markedly inhibited the ability of the latter to induce cell death and LDH release (Fig. 6D). Furthermore, this inhibitory effect of SmCASP6 was significantly blocked by CASP6 inhibitor (Fig. 6E). These results indicated that SmCASP6 had a destructive effect on both the unactivated SmGSDMEa and the activated SmGSDMEa (SmGSDMEa-NT_262_).

### SmGSDMEb is cleaved by SmCASP8 but unable to induce cell death

SmGSDMEb was specifically cleaved by rSmCASP8, and the cleavage was abolished when the D246 of SmGSDMEb was mutated to alanine (Fig. S3), implying that _243_FEVD_246_ is the recognition site of SmCASP8. HEK293T cells transfected with mCherry-tagged SmGSDMEb-FL, -NT and -CT showed abundant expression of the SmGSDMEb variants, but the cells exhibited no apparent alteration in morphology or LDH release (Fig. S4).

### *V. harveyi* infection promotes the production and activation of SmGSDMEa, but not SmGSDMEb, in turbot

**Fig. 7:**
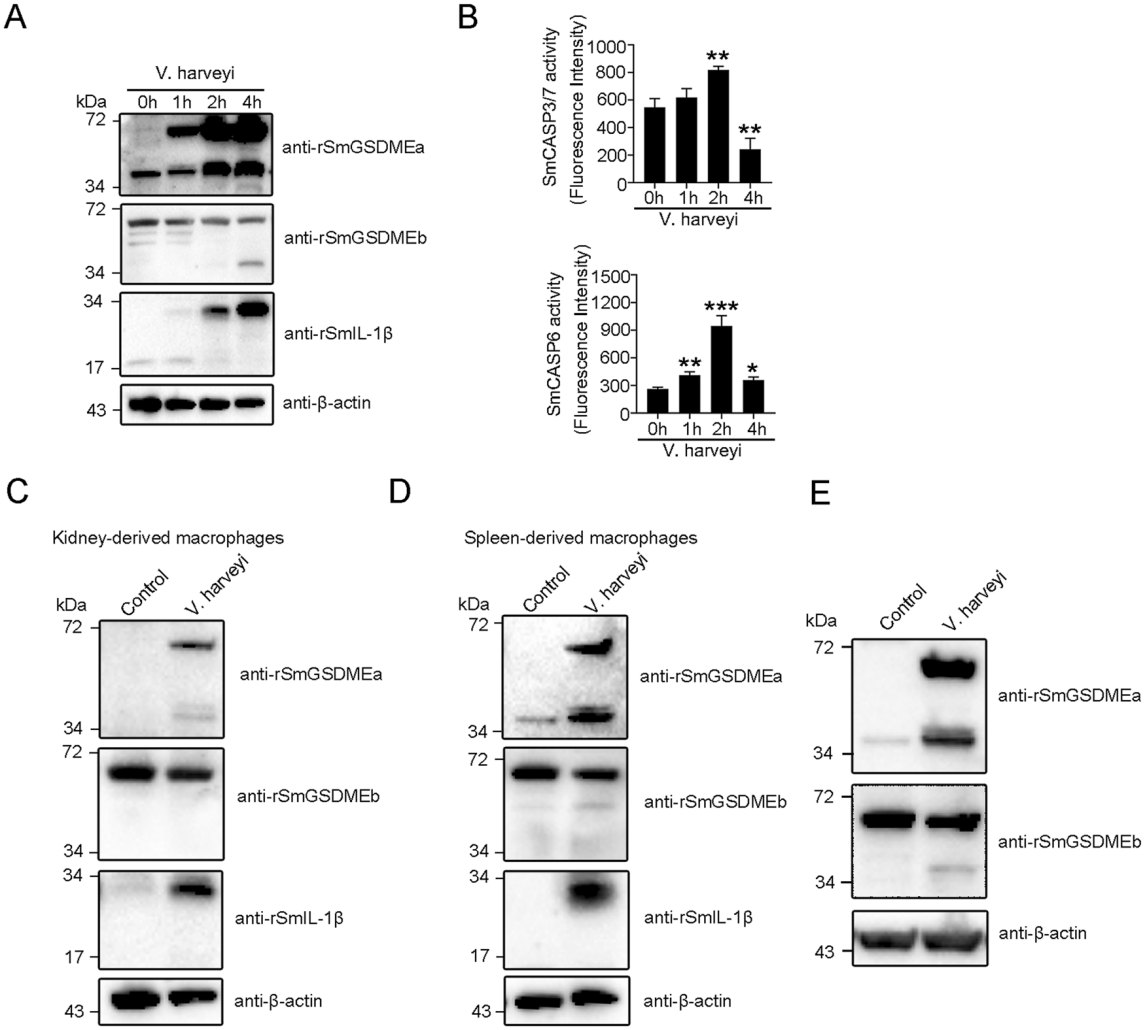
*Vibrio harveyi* infection induces SmGSDMEa production and activation. **A**, **B** Turbot peritoneal macrophages were treated with *V. harveyi* for different hours. The culture supernatant and cell lysate were immunoblotted with antibodies against rSmGSDMEa/b, rSmIL-1β, or β-actin and assayed for SmCASP3/7/6 activity. Values are shown as means ± SD, *n* = 3. ***P < 0.001; **P < 0.01; *P < 0.05. **C**, **D** Turbot macrophages from kidney and spleen were infected with *V. harveyi* for 4 h, and the culture supernatant and cell lysates were subjected to immunoblot as above. **E** Peritoneal macrophages from *V. harveyi*-infected turbot were immunoblotted with antibodies against rSmGSDMEa/b or β-actin.

To examine the effect of *V. harveyi* infection on SmGSDMEa/b production and activation in turbot cells, a series of cellular infections were performed. The results showed that following *V. harveyi* infection, turbot peritoneal macrophages exhibited strong induction and cleavage of SmGSDMEa in a time-dependent manner, accompanied with the production of abundant pro-IL-1β (Fig. 7A). In contrast, SmGSDMEb protein remained unchanged during infection and was only weakly cleaved in the late stage of infection (Fig. 7A). In accordance with the massive cleavage of SmGSDMEa, SmCASP3/7 in the infected cells was significantly activated at 2 h post-infection (hpi) and inactivated at 4 hpi, while SmCASP6 was significantly activated at 1 hpi, peaked at 2 hpi, and declined at 4 hpi (Fig. 7B). Similar results were obtained with the infection of spleen and head kidney macrophages (Fig. 7C, D). In line with these observations, in vivo infection showed that, unlike uninfected fish which exhibited very low level of SmGSDMEa, fish infected with *V. harveyi* exhibited dramatic inductions of the SmGSDMEa and its cleavage (Fig. 7E). On the contrary, SmGSDMEb was abundant in uninfected fish, and bacterial infection had no apparent effect on SmGSDMEb induction but induced a relatively low level of SmGSDMEb cleavage (Fig. 7E).

### SmCASP3/7 activity is required for optimal bacterial clearance in turbot

**Fig. 8:**
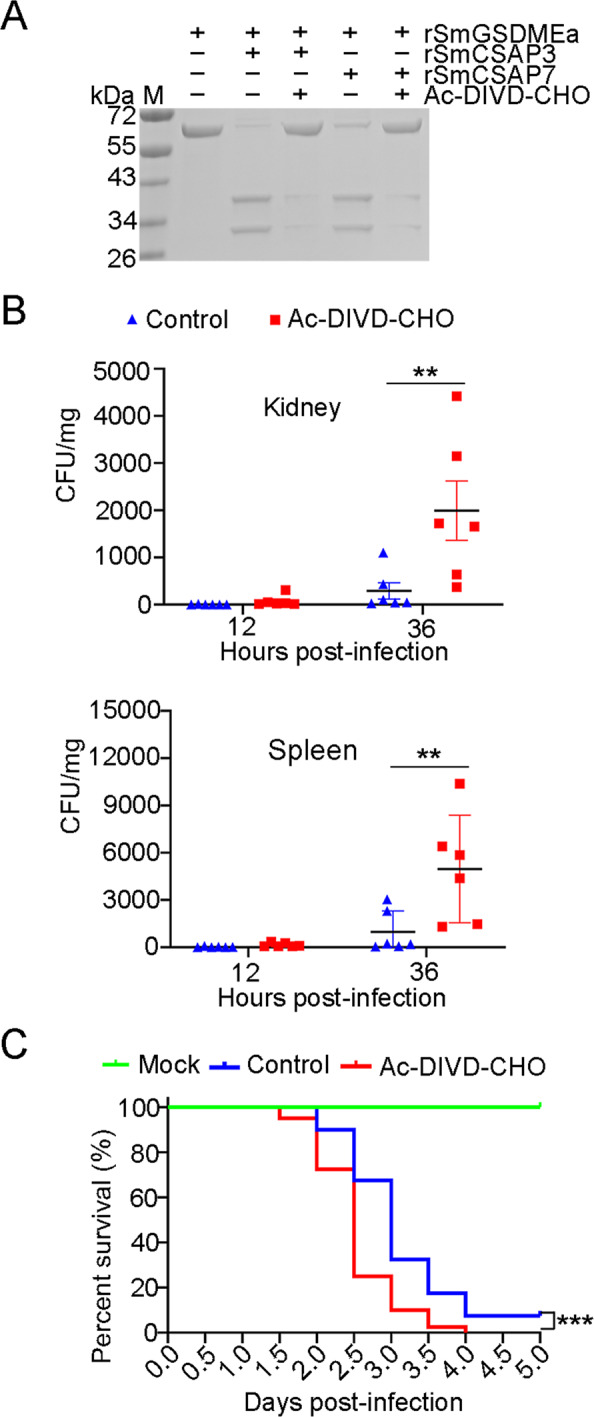
Interfering with the activity of SmCASP3/7 in turbot promotes *Vibrio harveyi* infection. **A** rSmGSDMEa was treated with rSmCASP3/7 in the presence or absence of Ac-DIVD-CHO. The samples were then analyzed by SDS-PAGE. **B** Turbot were infected with *V. harveyi* in the presence or absence (control) of Ac-DIVD-CHO for different hours, and bacterial loads (shown as Colony Forming Unit, CFU) in kidney and spleen were determined. Values are shown as means ± SD, *n* = 6. **P < 0.01. **C** Turbot in two groups were infected with *V. harveyi* in the presence or absence (control) of Ac-DIVD-CHO. The mock infection group was treated with PBS. The fish were monitored daily for mortality. The significance between the survivals of Ac-DIVD-CHO-treated fish and control fish was determined with logrank test. *n* = 40 ***P < 0.001.

To examine the importance of the SmCASP3/7-SmGSDMEa axis in the immune defense against bacterial infection, a tetrapeptide inhibitor of SmCASP3/7, Ac-DIVD-CHO, was synthesized based on the SmCASP3/7 recognition motif in SmGSDMEa. Ac-DIVD-CHO could effectively block the cleavage of SmGSDMEa by SmCASP3/7 (Fig. 8A). When turbot were infected with *V. harveyi* in the presence of Ac-DIVD-CHO, the bacterial loads in kidney and spleen were significantly increased (Fig. 8B). Consistently, the survival of turbot in the presence of Ac-DIVD-CHO was significantly decreased (Fig. 8C).

## Discussion

Release of the pore-forming NT fragment from the auto-inhibitory C terminus is critical to GSDM-executed pyroptosis. In human and mouse, the apoptotic CASP3 specifically cleaves GSDME at the DMPD motif in the inter-domain linker region, and the liberated NT domain induces apoptosis-to-pyroptosis switch [8, 9, 30]. It should be noticed that another mammalian apoptotic caspase, CASP7, which shares the same substrate cleavage specificity with CASP3, does not cleave human and mouse GSDME [8, 30]. In contrast, we found that in turbot, both SmCASP3 and SmCASP7 were able to cleave SmGSDME at the same inter-domain DIVD site to generate the pyroptosis-inducing NT fragment. These results suggest that in teleost, pyroptosis may be activated through multiple signaling pathways via different caspases. In addition to our observation in this study, this hypothesis can also find support in previous studies. For example, in zebrafish *Danio rerio*, GSDMEa-mediated pyroptosis occurred when GSDMEa was cleaved by CASP3, while GSDMEb-dependent pyroptosis was activated by caspy2 during *Edwardsiella tarda* infection [13, 14]. Unlike mammals that have a group of pyroptosis-inducing GSDM members, teleost have only GSDME to execute pyroptosis [11]. Hence, activation of GSDME via multiple signaling pathways might enable GSDME-mediated pyroptosis to participate in different situations, thus facilitating teleost to cope with different stresses.

In addition to activate GSDM, proteolytic cleavage by caspase as well as other proteases can also inactivate some GSDM [31, 32]. It has been shown that CASP1/4/5-mediated, GSDMD-induced pyroptosis was abolished by CASP3/7 cleavage at a DAMD site within the N-terminus of GSDMD, which destroyed the functional NT domain and caused apoptosis instead of pyroptosis [31]. Since the DAMD motif is evolutionarily conserved in mammalian GSDMD, including that of *Homo sapiens*, *Pan troglodytes*, *Bos taurus*, *Mus musculus*, and *Rattus norvegicus*, it is possible that this type of negative regulation is commonly present in mammals [31]. In GSDMB-mediated pyroptosis, the serine protease granzyme A cleaved GSDMB at two neighboring sites after K229 and K244 to produce the pyroptosis-inducing NT fragment, whereas CASP3/6/7 cleaved after the consensus site DNVD within the NT domain, thus impairing the intact architecture of the NT fragment [32, 33]. Several proteases are known to cleave GSDME and activate pyroptosis in human, mouse, and fish. However, negative regulation of GSDME-mediated pyroptosis by protease has not yet been reported. In the present study, we found that SmGSDMEa was cleaved by SmCASP3/7 and SmCASP6, resulting in two different NT fragments (NT_262_ and NT_202_). While NT_262_ was an effective pyroptosis inducer, NT_202_ was incapable of inducing pyroptosis. This observation is in line with the previous reports that the integrity of the NT domain is necessary for GSDM to form pores on the cytoplasmic membrane, and that truncation of the C terminus of human GSDME-NT to less than 235 residues impaired its pyroptosis-inducing capacity [9]. The cryo-electron microscopy structure of mouse GSDMA3 transmembrane ring pore suggested an important role of the C terminus of NT in the interface interaction of the pore globular domain oligomerization [7]. In our study, we also observed that the NT_262_ produced by CASP3/7 cleavage could be further cleaved by SmCASP6 into the truncated, loss-of-function NT_202_, thus nullifying the activation effect of SmCASP3/7. Together these results indicate that SmGSDMEa is under opposing regulations by SmCASP3/7 and SmCASP6, which may balance the activity of SmGSDMEa to a level most beneficial to the host.

*V. harveyi* is a marine bacterial pathogen with a broad host range, including fish, coral, shrimp, and mollusc. In flounder *Paralichthys olivaceus*, *V. harveyi* infection activated CASP3/7 and induced apoptosis [28, 34], but the mechanism underlying *V. harveyi*-induced programmed necrotic cell death is unclear. In our study, we found that *V. harveyi* infection markedly increased the expression of SmGSDMEa at both mRNA and protein levels, implying that SmGSDMEa is a bacteria-inducible immune factor. Moreover, the infection significantly increased CASP3/7 activity and caused SmGSDMEa cleavage into the pyroptosis-inducing NT_262_ in turbot tissues, which was in agreement with the pyroptotic cell death observed in the infected fish. Contrary to SmGSDMEa, SmGSDMEb expression did not change obviously at the protein level and even decreased at the mRNA level after *V. harveyi* infection. At the late stage of infection, a slight cleavage of SmGSDMEb was detected. However, since the cleaved SmGSDMEb exhibited no apparent harmful effect on the cells, we speculate that, unlike SmGSDMEa, SmGSDMEb is probably not a significant player in anti-bacterial immunity. During *V. harveyi* infection, blockage of CASP3/7 activity significantly increased bacterial dissemination and decreased host survival, suggesting an immunoprotective role of the pyroptosis mediated by the CASP3/7-SmGSDMEa axis. During the infection, we also observed increased production of IL-1β in turbot, however, no mature IL-1β was detected, suggesting that CASP3/7 and CASP6 may not be involved in processing pro-IL-1β.

**Fig. 9:**
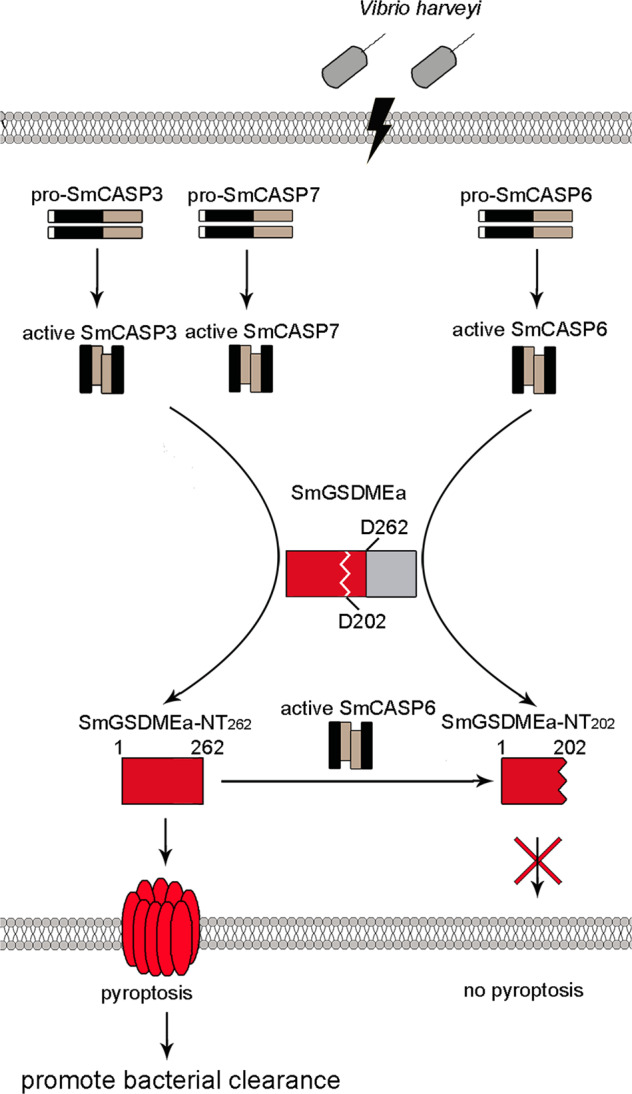
A proposed model of the bi-directional regulation of the activation and anti-bacterial effect of GSDME-mediated pyroptosis in the course of *Vibrio harveyi* infection in turbot. *V. harveyi* infection causes the activation of SmCASP3/7 and SmCASP6, which then cleave SmGSDMEa at D262 and D202, respectively, resulting in two N-terminal (NT) fragments – the pyroptotic NT_262_ and the non-pyroptotic NT_202_, respectively. NT_262_ subsequently induces pyroptosis that leads to effective *V. harveyi* clearance. NT_262_-mediated pyroptosis is negatively regulated by SmCASP6, which destroys the pyroptotic activity of NT_262_ by cleaving it into NT_202_.

Collectively, our study revealed the existence of a bi-directional regulation mechanism that governs the activation of GSDME-mediated pyroptosis via caspase in teleost (Fig. 9), and that teleost CASP3/7 and 6, which have long been considered apoptotic executors in mammals, act as direct pyroptosis regulators in positive and negative manners, respectively. Our results also revealed a regulatory and functional difference between teleost GSDMEa and GSDMEb in the course of bacterial infection, and highlighted the role of GSDMEa as an important participant in fish immune defense. These findings add new insights into the function of GSDME and CASP of lower vertebrate.

## Materials and methods

### Animal

Clinically healthy teleost (*Scophthalmus maximus*) were purchased from a local fish farm. The fish were maintained at 20 °C in aerated seawater as reported previously [34]. For experiments involving tissue collection, the fish were euthanized with an overdose of tricaine methane sulfonate (Sigma, St. Louis, MO). The live animal studies were approved by the Ethics Committee of the Institute of Oceanology, Chinese Academy of Sciences.

### Cell culture and transfection

HEK293T cells (American Type Culture Collection) were grown in Dulbecco’s modified Eagle’s medium supplemented with 10% (v/v) fetal bovine serum (Gibco, Renfrewshire, UK) at 37 °C with 5% CO_2_. *Vibrio harveyi* [35] was cultured in Luria Bertani broth (LB) medium at 28 °C with shaking (180 rpm). Lipofectamine 3000 Transfection Reagent (Invitrogen, USA) was used for transient transfection of plasmid into HEK293T cells. For gene overexpression, pmCherry-N1 or pCMV-C-Myc (Clontech, Mountain View, CA, USA) expressing SmGSDMEa/b variants (full length and NT/CT fragments) and SmCASP variants were introduced into HEK293T cells by transfection as above. The primers used are listed in Table S1.

### Sequence and phylogenetic analysis

For sequence alignment, gasdermin sequences were aligned using Clustal W program (www.ebi.ac.uk/clustalw/) [36]. The picture was generated using ESPript 3.0 (http://espript.ibcp.fr/ESPript/cgi-bin/ESPript.cgi) [37]. For phylogenetic analysis, 17 GSDMEa and 67 GSDMEb were collected from NCBI Orthologs as references. The sequences were aligned by Clustal Omega [38] and phylogenetic tree was subsequently constructed via IQ-TREE 2 using maximum likelihood method with 1000 bootstrap replications [39]. The phylogenetic analysis were conducted with the Best-fit substitution model JTT + F + R6 according to Bayesian Information Criterion. The final presented tree was edited with iTOL (https://itol.embl.de/) [40].

### Gene cloning and sequence mutagenesis

The codon-optimized protein coding sequences (CDS) of SmCASP3/6/7/8 and SmGSDMEa/b were amplified by PCR. The CDS of SmGSDMEa-NT_262_, SmGSDMEa-NT_202_, SmGSDMEa-CT_262_, SmGSDMEa-CT_202_, SmGSDMEb-NT and SmGSDMEb-CT were subcloned from the above cloning sequence. Site-directed mutations of SmGSDMEa Asp202 to Ala (D202A), Asp259 to Ala (D259A), Asp262 to Ala (D262A), and mutation of SmGSDMEb Asp246 to Ala (D246A) were performed using the Hieff Mut Site-Directed Mutagenesis Kit (Yeasen, Shanghai, China). The recombinant plasmid was introduced into Trelief 5α (Tsingke Biological Technology, Beijing, China) by transformation. The mutations were verified by sequencing analysis. The primers used are listed in Table S1.

### Edman sequencing

Edman sequencing was performed as previously reported [12]. Briefly, rSmGSDMEa was incubated with rSmCASP6 at 26 °C for 4 h, followed by 12% SDS-PAGE to separate the cleaved fragments. The proteins were transferred onto polyvinylidene difluoride (PVDF) membrane (Millipore, MA, USA) and stained with 0.1% (w/v) Ponceau S for 10 min. The cleaved bands were cut out and subjected to Edman degradation in a PPSQ-33A automated protein sequencer (Shimadzu, Kyoto, Japan).

### Preparation of recombinant proteins

Recombinant proteins of SmGSDMEa/b and active forms of SmCASP3/6/7/8 were prepared from *Escherichia coli* as described previously [3, 12]. Briefly, recombinant pET30a (+) vectors (Novagen, Madison, WI, USA) expressing SmGSDMEa/b and SmCASP3/6/7/8 were introduced into *E. coli* Transetta (DE3) (TransGen, Beijing, China) by transformation. The cells were cultured to OD_600_ 0.6 at 37 °C in LB broth. Isopropyl-β-D-thiogalactopyranoside (IPTG) was added to the culture at a final concentration of 0.2 mM, followed by incubation at 15 °C for 20 h. The bacteria cells were harvested by centrifugation, and lysed on ice by ultrasonication. The supernatant was collected after centrifugation at 12,000 rpm at 4 °C for 1 h. The recombinant protein was purified using a nickel-nitrilotriacetic acid (Ni-NTA) column (GE Healthcare, Uppsala, Sweden). To prepare recombinant turbot IL-1β (rSmIL-1β), pET30a expressing SmIL-1β was introduced into *E. coli*, and the expression of the recombinant protein was induced by IPTG as above. The cells were ultrasonically lysed, and the pellet of the cells was used to purify the protein under denaturing condition as reported previously [41]. All purified proteins were dialyzed three times for 24 h in PBS at 4 °C and concentrated using Ultra free centrifugal filters (Millipore, MA, USA). The protein concentration was determined using the BCA Protein Assay Kit (Pierce Chemical, Rockford, IL, USA).

### Preparation of polyclonal antibodies against rSmGSDMEa/b and rSmIL-1β

Mouse polyclonal antibodies against rSmGSDMEa/b and rSmIL-1β were prepared as reported previously [12]. Briefly, 50 μg of recombinant protein in 500 μl PBS was emulsified in 500 μl complete Freund’s adjuvant (Sigma-Aldrich, Madrid, Spain). BALB/c mice (8 weeks) were immunized by multipoint subcutaneous implantation, followed by three boosts with the protein emulsified in incomplete Freund’s adjuvant (Sigma-Aldrich, Madrid, Spain) at 1-week intervals. Blood was collected, and serum was separated by centrifugation at 2000 *g*, 4 °C for 15 min. Anti-rSmGSDMEa/b and anti-rSmIL-1β immunoglobulin G (IgG) were further purified using immobilized protein G (Thermo Fisher Scientific, South Logan, UT, USA) according to the manufacturer’s instruction.

### Lactate dehydrogenase (LDH) assay

Cell death was estimated by assaying the activity of LDH released into the cell culture supernatant using the CytoTox96 LDH release kit (Promega, Leiden, Netherlands) according to the manufacturer’s protocol. The percent cytotoxicity was calculated using the following formula: percent cytotoxicity = 100 × (experimental sample – culture medium background)/ (maximum LDH release − culture medium background).

### Microscopy

To examine the morphology of pyroptotic cells, the cells were plated on 24-well plate (Costar, Corning, NY, USA) at about 60% confluency and subjected to the indicated treatment. For cell membrane and nuclear staining, the cells transfected with pCMV-c-Myc vector expressing SmGSDMEa-NT were stained with DiO (Solarbio, Beijing, China) for 30 min and propidium iodide (PI) (Invitrogen, Carlsbad, CA, USA) for 5 min. The cells transfected with the backbone vector were prefixed with 4% paraformaldehyde for 10 min and then stained with DiO and PI as above. To video the cell death process, the cells were seeded into 35 mm glass-bottom culture dishes (Nest Scientific, Rahway, NJ) at about 60–70% confluency. The bright-field and fluorescent views of the cells and the process of cell death were recorded using a Carl Zeiss LSM 710 confocal microscope (Carl Zeiss, Jena, Germany).

### CASP activity assay

To determine the cleavage specificity and activity of rSmCASPs, the proteins were each incubated with various colorimetric substrates as described previously [12], followed by monitoring the released ρNA at OD405 nm. The CASP activities in turbot cells were determined as reported previously [12]. Briefly, cell lysates were buffered in a 100 μl reaction system [50 mM Hepes (pH 7.5), 3 mM EDTA, 150 mM NaCl, 0.005% (v/v) Tween 20, and 10 mM dithiothreitol (DTT)]. The CASP substrates, Ac-YVAD-AFC, Ac-YDQQD-AFC, Ac-DEVD-AFC, Ac-VEID-AFC, Ac-IETD-AFC, and Ac-LEHD-AFC (MedChem Express, NJ, USA), were each added to the cell lysates at the final concentration of 200 μM. Fluorescence was measured every 10 min for 60 min with a BioTek Synergy HT plate reader (BioTek Instruments, VT, USA).

### SmGSDME cleavage by CASPs

The GSDME cleavage assay was performed as describe previously [3, 12]. Briefly, rSmGSDMEa/b was incubated with 1 U of rHsCASP1, 2, 3, 6, 7, 8, or 9 (Enzo Life Sciences, Villeurbanne, France) at 37 °C for 2 h in a 50 μl reaction system containing 50 mM Hepes (pH 7.5), 3 mM EDTA, 150 mM NaCl, 0.005% (v/v) Tween 20, and 10 mM DTT. To examine rSmGSDMEa/b cleavage by rSmCASPs, rSmGSDMEa/b or its mutant was incubated with rSmCASPs at 26 °C for 2 h in a 50 μl reaction system. The cleaved fragments were separated by SDS-PAGE, followed by Coomassie Brilliant Blue (CBB) R250 staining and immunoblotting with antibody against His tag.

### SmGSDME/SmIL-1β production and cleavage in *V. harveyi-*infected turbot cells

Turbot kidney- and spleen-derived macrophages were isolated as reported previously [42]. In brief, the tissues were gently triturated and passed through a 40 μm sterile nylon cell strainer (Biosharp, Anhui, China), followed by 51% percoll (GE Healthcare, Uppsala, Sweden) gradient separation. The harvested cells were cultured in L15 medium (Sigma-Aldrich, Madrid, Spain) at 27 °C for 1 h. The non-adherent cells were washed off, and the adherent macrophages were infected with *V. harveyi* at a multiplicity of infection (MOI) of 1:1. At 1 h, 2 h, and 4 h post-infection, the cells and supernatants were collected after centrifugation at 600 *g* for 5 min. Peritoneal macrophages (PMs) from *V. harveyi*-infected turbot were isolated with the peritoneal lavage method according to a previous report [12]. Briefly, turbot (~500 *g*) were injected intraperitoneally (i.p.) with 50 μg ultrapure LPS (InvivoGen, San Diego, CA, USA). At 4 d after injection, the fish were inoculated with 10^6^ CFU *V. harveyi* via i.p. injection. Macrophages were collected from the peritoneal cavity of the fish at 2 h post infection (hpi) by washing with ice-cold PBS three times. The cells were pelleted by centrifugation at 600 *g* for 5 min. To examine SmGSDME/SmIL-1β production and cleavage in the prepared turbot cells, the cells were lysed in RIPA Lysis Buffer (Beyotime, Shanghai, China). The supernatants were collected and precipitated using TCA (15% final concentration) at 4 °C overnight. The precipitates were collected after centrifugation at 15,000 *g* at 4 °C for 30 min and washed with ice-cold acetone. The precipitated proteins were mixed with cell lysates in 1:1 ratio, and the mixture was subjected to SDS-PAGE. The proteins were then transferred to nitrocellulose membranes (Millipore, MA, USA) and immunoblotted with antibodies against rSmGSDMEa/b, rSmIL-1β, or β-actin (Abcam, Cambridge, MA, USA). The immune reactive protein bands were visualized using an ECL kit (Sparkjade Biotechnology Co. Ltd., Shandong, China).

### Bacterial dissemination in fish tissues

Turbot were randomly divided into two groups (15 fish/group). One group was intraperitoneally (i.p.) injected with 10 μg Ac-DIVD-CHO (Science Peptide Biological Technology Co., Ltd, shanghai, China), while the control group was injected with sterile PBS. At 8 h post injection, the fish were inoculated with 2 × 10^6^ CFU *V. harveryi* via intramuscularly (i.m.) injection. At 12 and 36 hpi, kidney and spleen were collected and homogenized in sterile PBS. The homogenates were serially diluted in PBS, and the dilutions were plated on LB plates. After incubation at 28 °C for 18 h, the colonies on the plates were counted.

### Fish survival after bacterial infection

Turbot were randomly divided into three groups (40 fish/group) named A, B, and C. Group A was administered with 10 μg Ac-DIVD-CHO via i.p. injection, and the other two groups were injected similarly with PBS. At 8 h post injection, groups A and B were infected with *V. harveyi* (2.0 × 10^6^ CFU/fish) via i.m. injection, while group C (mock infection group) was injected similarly with PBS. The fish were monitored daily for mortality.

### Quantitative real time RT-PCR (qRT-PCR)

qRT-PCR was performed as reported previously [43]. Briefly, tissues (intestine, head kidney, blood, liver, spleen, heart, gill, muscle, and brain) were taken aseptically from five turbot and used for total RNA extraction with Trizol reagent (Invitrogen, Carlsbad, CA, USA). cDNA synthesis was performed with First Strand cDNA Synthesis Kit (ToYoBo, Japan) according to the manufacturer’s protocol. qRT-PCR was carried out in an Eppendorf Mastercycler (Eppendorf, Hamburg, Germany) using the SYBR ExScript qRT-PCR Kit (Vazyme Biotech Co. Ltd., Nanjing, China). To examine gene expression in *V. harveryi*-infected fish, turbot were divided randomly into two groups (25 fish/group) and injected intramuscularly with *V. harveryi* (2.0 × 10^6^ CFU/fish) or PBS. At 6, 12, 24, and 48 hpi, tissues were collection (five fish/time point). cDNA preparation and qRT-PCR were performed as above. The mRNA levels of the target genes were normalized to that of β-actin [44]. The PCR primers are listed in Table S1.

### Statistical analysis

All statistical analyses were performed with GraphPad Prism 7 (www.graphpad.com/) software. Student’s *t-*test and one-way analysis of variance (ANOVA) were used for comparisons between groups. Log-rank was used for the analysis of fish survival. Statistical significance was defined as *P* < 0.05.

## Supplementary information

Checklist

Supplemental Materials

Table S1

Fig. S1

Fig. S2

Fig. S3

Fig. S4

Movie S1

Movie S2

## Data Availability

All data in the paper are present in the paper or the Supplementary Materials.
