# Supplementary material for: GSDMEa-mediated pyroptosis is bi-directionally regulated by caspase and required for effective bacterial clearance in teleost
Source: Cell Death Dis. 2022 May 24;13(5):491. doi: 10.1038/s41419-022-04896-5 (PMC9130220; doi:10.1038/s41419-022-04896-5)
Supplement: Supplementary file 2 — Supplemental Materials [file 41419_2022_4896_MOESM2_ESM.docx]

**Supplementary materials**

**The file include:**

Table S1. Primers used in this study.

Fig. S1. Phylogenetic analysis of SmGSDMEa and SmGSDMEb.

Fig. S2. The expression of SmGSDMEa/b in turbot tissues.

Fig. S3. rSmGSDMEb is cleaved by rSmCASP8.

Fig. S4. SmGSDMEb and its NT and CT regions fail to induce cell death.

Legends for Movies S1 and S2.

Movie S1 (gif format). SmGSDMEa-NT_262_ induces pyroptosis of HEK293T cells.

Movie S2 (gif format). SmGSDMEa-NT_202_ is unable to induce cell death.

**Table S1. Primers used in this study.**

| Primer | Sequence (5'→3') |
| --- | --- |
| Gene cloning and sequence mutagenesis  M13 forward  M13 reverse  SmGSDMEa forward  SmGSDMEa reverse  SmGSDMEb forward  SmGSDMEb reverse  SmCASP3 forward  SmCASP3 reverse  SmCASP6 forward  SmCASP6 reverse  SmCASP7 forward  SmCASP7 reverse  SmCASP8 forward  SmCASP8 reverse  SmGSDMEa-D259A forward  SmGSDMEa-D259A reverse  SmGSDMEa-D262A forward SmGSDMEa-D262A reverse  SmGSDMEa-D202A forward  SmGSDMEa-D202A reverse  SmGSDMEb-D246A forward  SmGSDMEb-D246A reverse  Preparation of recombinant proteins  T7 promoter primer  T7 terminator primer  SmGSDMEa forward  SmGSDMEa reverse  SmGSDMEb forward  SmGSDMEb reverse  SmCASP3 forward  SmCASP3 reverse  SmCASP6 forward  SmCASP6 reverse  SmCASP7 forward  SmCASP7 reverse  SmCASP8 forward  SmCASP8 reverse  Gene overexpression  SmGSDMEa forward  SmGSDMEa reverse  SmGSDMEb forward  SmGSDMEb reverse  SmGSDMEa-NT_262_ reverse  SmGSDMEa-CT_262_ forward  SmGSDMEa-NT_202_ reverse  SmGSDMEa-CT_202_ forward  SmGSDMEb-NT reverse  SmGSDMEb-CT forward  SmCASP3 forward  SmCASP3 reverse  SmCASP6 forward  SmCASP6 reverse  SmCASP7 forward  SmCASP7 reverse  qRT-PCR  SmGSDMEa forward  SmGSDMEa reverse  SmGSDMEb forward  SmGSDMEb reverse  β-actin forward  β-actin reverse | TGTAAAACGACGGCCAGT  CAGGAAACAGCTATGACC  ATGTTTTCCAAGGCCACTGCC  CTCCGGCCCGTTGCACAA  ATGTTTGCCACAGCGACCA  AACACAGACGGTCAATGAGG  ATGTCGGTGAATGGACCC  AGGAGAGAAATACATCTCTTTAGTCAG  ATGTCCAACACGGCGG  CTTTTTTGGTCGGAAGTATAGTTTCT  ATGGCCGCGGGAGCT  GTTGAAATACAGTTCCTTCGTCAG  ATGGATAGACTGAAGCTGTCCA  ATGGATAGACTGAAGCTGTCCA  CCCCGATCCCTCAAATGCTATTGTGGAT  GCATTTGAGGGATCGGGGGACGGACAGGA  TCAAATGATATTGTG GCT GGCAAGTTC  GCCACAATATCATTTGAGGGATCGGGGG  CAACATCGAGGTGGCCAGTGATGTCTC  TGTAGCTCCACCGGTCACTACAGAGTA  GGAGGTTTCGAAGTCGCCAGCTCTGCTAAG  CCAAAGCTTCAGCGGTCGAGACGATTCTTT  TAATACGACTCACTATAGGG  TGCTAGTTATTGCTCAGCGG  TAAGAAGGAGATATACATATGATGTTTTCCAAGGCCACTGCC  GTGGTGGTGGTGGTGCTCGAG CTCCGGCCCGTTGCACAA  TAAGAAGGAGATATACATATG ATGTTTGCCACAGCGACCA  GTGGTGGTGGTGGTGCTCGAGAACACAGACGGTCAATGAGG  TAAGAAGGAGATATACATATGATGTCGGTGAATGGACCC  GTGGTGGTGGTGGTGCTCGAGAGAAATACATCTCTTTAGTCAG  GATATC ATGTCTAACATGGCGGAAGAC GATATC CTTTTTTGGTCGGAAGTATAGTTTCT  TAAGAAGGAGATATACATATGATGGCCGCGGGAGCT  GTGGTGGTGGTGGTGCTCGAGATGGCCGCGGGAGCT  GGAATTCCATATGATGCATGATGAGGCGGAGTACTAC  CCGGAATTC TACGAACTTGAGGACAAGCTTCT  CATGGTGGCGACCGGTGGATCATGTTTTCCAAGGCCACTGCC  TCAGATCTCGAGCTCAAGCTTCTCCGGCCCGTTGCACAA  CATGGTGGCGACCGGTGGATCATGTTTGCCACAGCGACCA  TCAGATCTCGAGCTCAAGCTTAACACAGACGGTCAATGAGG  CATGGTGGCGACCGGTGGATCATCCACAATATCATTTGAGGGATC  TCAGATCTCGAGCTCAAGCTTATGGGCAAGTTCAATGGGGAG  CATGGTGGCGACCGGTGGATCGTCCACCTCGATGTTGTTGC  TCAGATCTCGAGCTCAAGCTTATGAGTGATGTCTCATTGGAGATCC  CATGGTGGCGACCGGTGGATCGTCGACTTCGAAACCTCCGTT  TCAGATCTCGAGCTCAAGCTTATGTCTGCTAAGAAAGGACTGTTG  ATCCAAGCTTCTGCAGGAATTCATGTCGGTGAATGGACCC  TTTCTGCTCTCTAGACTCGAG AGAGAAATACATCTCTTTAGTCAG  ATCCAAGCTTCTGCAGGAATTCATGTCCAACACGGCGG  TTTCTGCTCTCTAGACTCGAGCTTTTTTGGTCGGAAGTATAGTTTCT  ATCCAAGCTTCTGCAGGAATTCATGGCCGCGGGAGCT  TTTCTGCTCTCTAGACTCGAG GTTGAAATACAGTTCCTTCGTCAG  TTTCACCCTCGGCAACTTGT  AGCCTTGGTGTCCAGCTTAC  CAATGTGTTGCAGCCACGAA  TGCGGTCCTTTCAAAGACGA  CGTGCGTGACATCAAGGAG  AGGAAGGAAGGCTGGAAGAG |

**Fig. S1. Phylogenetic analysis of SmGSDMEa and SmGSDMEb.** The phylogenetic tree was constructed using 17 fish GSDMEa (blue) and 67 fish GSDMEb (red) collected from NCBI orthologs. SmGSDMEa and SmGSDMEb are marked with blue and red stars, respectively.

**Fig. S2. The expression of SmGSDMEa/b in turbot tissues.** SmGSDMEa and SmGSDMEb expression in turbot tissues under normal physiological conditions was determined by qRT-PCR. Values are shown as means ± SD, n=3.

**
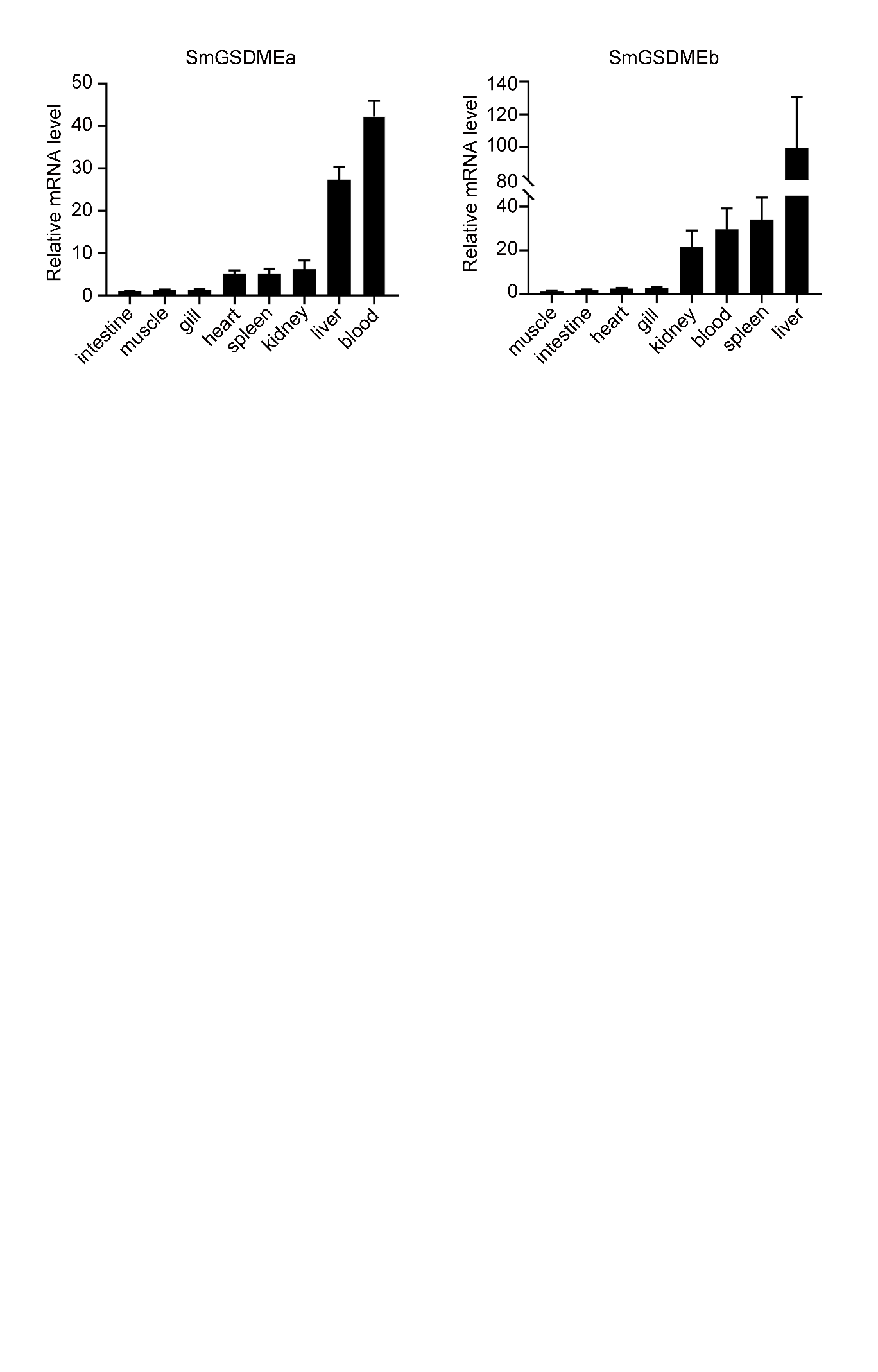
**

**Fig. S3. rSmGSDMEb is cleaved by rSmCASP8. (A)** rSmGSDMEb was treated with different units of rSmCASP8, and then subjected to SDS-PAGE. **(B)** rSmGSDMEb and its D246A mutant were treated with rSmCASP8 and then analyzed by SDS-PAGE. NT, N-terminal fragment; CT, C-terminal fragment.


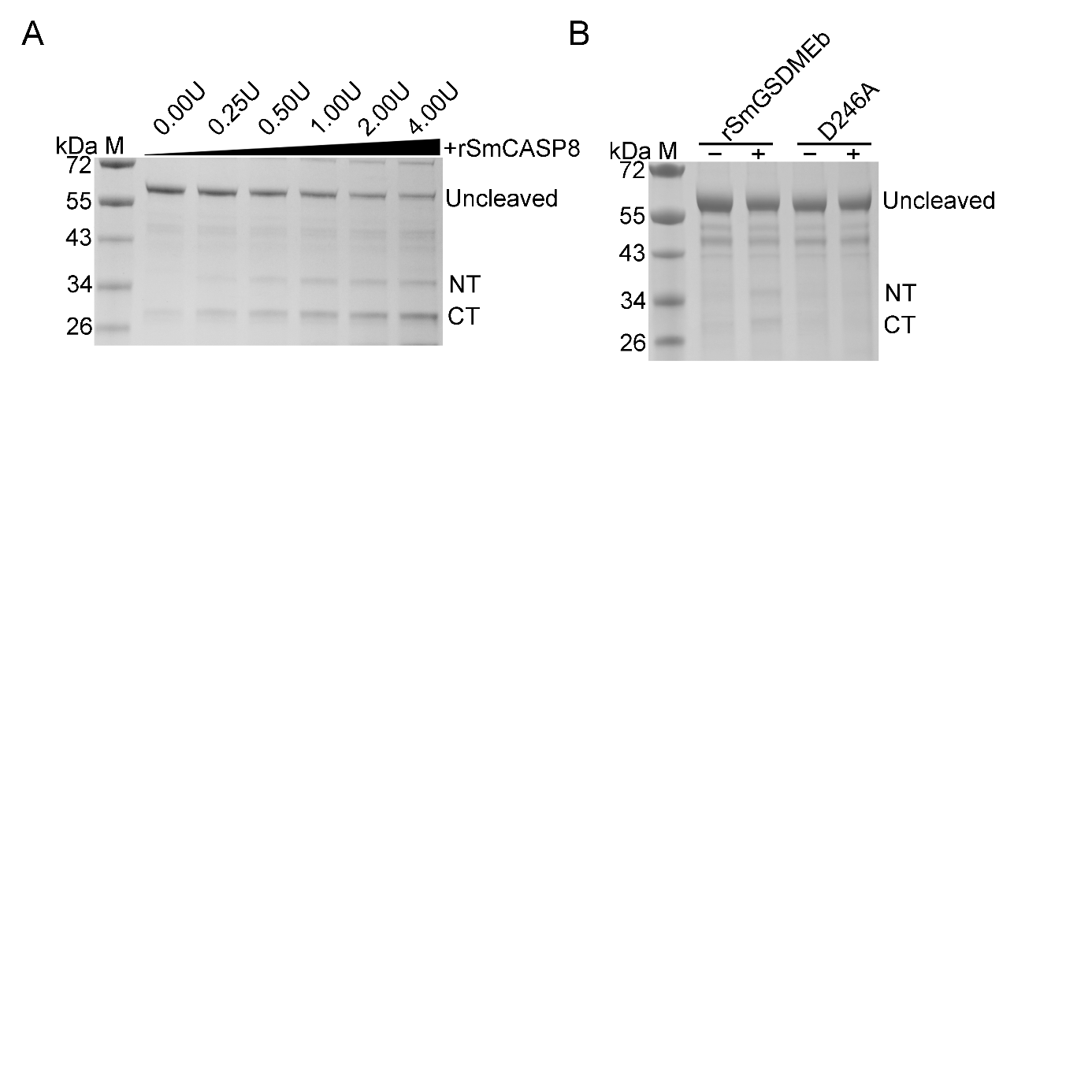


**Fig. S4. SmGSDMEb and its NT and CT regions fail to induce cell death. (A and B)** Fluorescence (A) and bright field (B) images of HEK293T cells transfected with the backbone vector or the vector encoding SmGSDMEb-FL, -NT, or -CT for 24 h. Scale bar, 50 μm. **(C)** LDH released from the above transfected cells was determined. Values are shown as means ± SD, n=3.

**
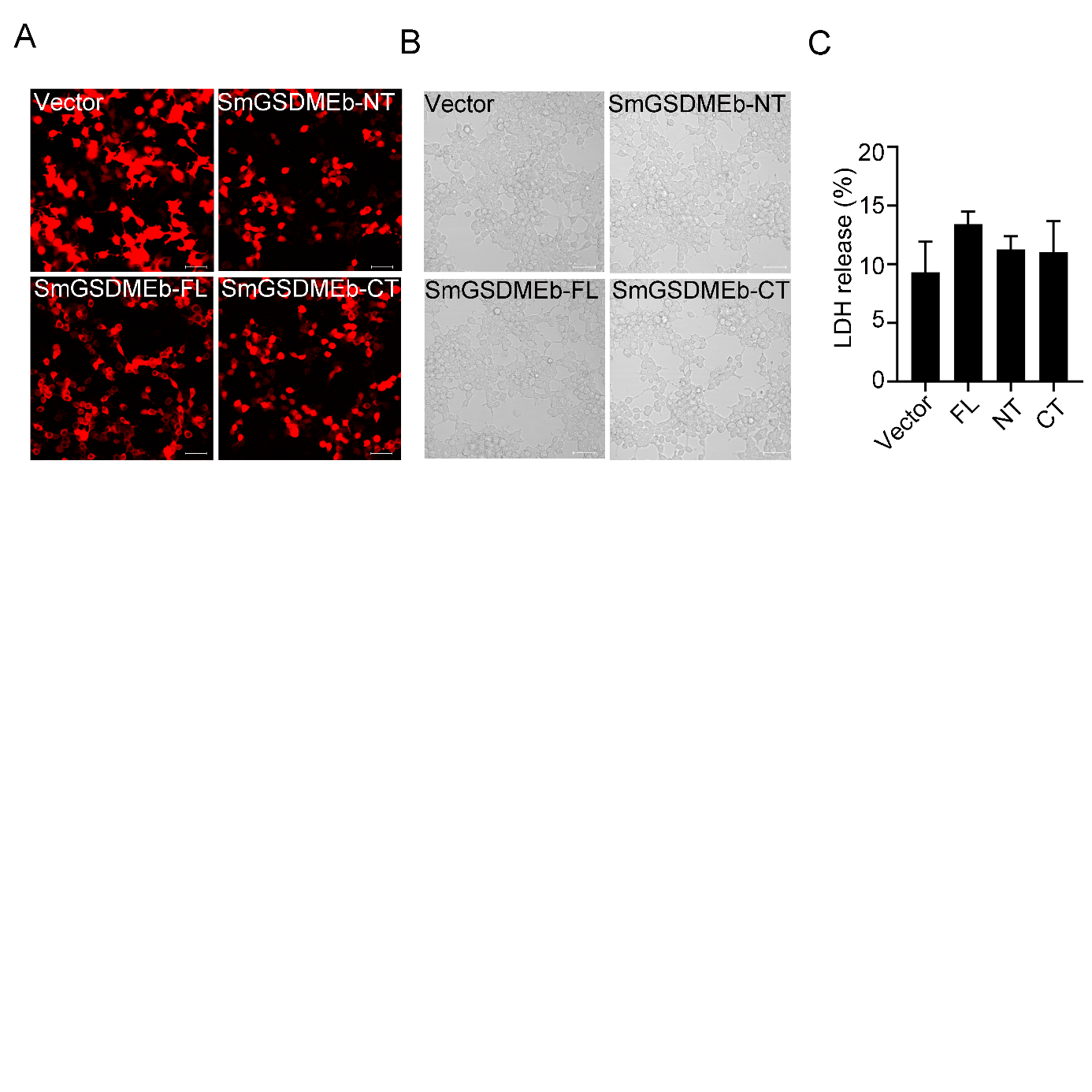
**

**Movie S1.** **SmGSDMEa-NT_262_ induces pyroptosis of HEK293T cells.**

HEK293T cells were transfected with the vector expressing mCherry-tagged SmGSDME-NT262, and time-lapse images of the cells were taken using a Carl Zeiss LSM 710 confocal microscope.

**Movie S2.** **SmGSDMEa-NT_202_ is unable to induce cell death.**

HEK293T cells were transfected with the vector expressing mCherry-tagged SmGSDME-NT202, and time-lapse images of the transfected cells were taken using a Carl Zeiss LSM 710 confocal microscope.
