# Supplementary material for: GSDMEa-mediated pyroptosis is bi-directionally regulated by caspase and required for effective bacterial clearance in teleost
Source: Cell Death Dis. 2022 May 24;13(5):491. doi: 10.1038/s41419-022-04896-5 (PMC9130220; doi:10.1038/s41419-022-04896-5)
Supplement: Supplementary file 3 — Table S1 [file 41419_2022_4896_MOESM3_ESM.docx]

**Table S1. Primers used in this study.**

| Primer | Sequence (5'→3') |
| --- | --- |
| Gene cloning and sequence mutagenesis  M13 forward  M13 reverse  SmGSDMEa forward  SmGSDMEa reverse  SmGSDMEb forward  SmGSDMEb reverse  SmCASP3 forward  SmCASP3 reverse  SmCASP6 forward  SmCASP6 reverse  SmCASP7 forward  SmCASP7 reverse  SmCASP8 forward  SmCASP8 reverse  SmGSDMEa-D259A forward  SmGSDMEa-D259A reverse  SmGSDMEa-D262A forward SmGSDMEa-D262A reverse  SmGSDMEa-D202A forward  SmGSDMEa-D202A reverse  SmGSDMEb-D246A forward  SmGSDMEb-D246A reverse  Preparation of recombinant proteins  T7 promoter primer  T7 terminator primer  SmGSDMEa forward  SmGSDMEa reverse  SmGSDMEb forward  SmGSDMEb reverse  SmCASP3 forward  SmCASP3 reverse  SmCASP6 forward  SmCASP6 reverse  SmCASP7 forward  SmCASP7 reverse  SmCASP8 forward  SmCASP8 reverse  Gene overexpression  SmGSDMEa forward  SmGSDMEa reverse  SmGSDMEb forward  SmGSDMEb reverse  SmGSDMEa-NT_262_ reverse  SmGSDMEa-CT_262_ forward  SmGSDMEa-NT_202_ reverse  SmGSDMEa-CT_202_ forward  SmGSDMEb-NT reverse  SmGSDMEb-CT forward  SmCASP3 forward  SmCASP3 reverse  SmCASP6 forward  SmCASP6 reverse  SmCASP7 forward  SmCASP7 reverse  qRT-PCR  SmGSDMEa forward  SmGSDMEa reverse  SmGSDMEb forward  SmGSDMEb reverse  β-actin forward  β-actin reverse | TGTAAAACGACGGCCAGT  CAGGAAACAGCTATGACC  ATGTTTTCCAAGGCCACTGCC  CTCCGGCCCGTTGCACAA  ATGTTTGCCACAGCGACCA  AACACAGACGGTCAATGAGG  ATGTCGGTGAATGGACCC  AGGAGAGAAATACATCTCTTTAGTCAG  ATGTCCAACACGGCGG  CTTTTTTGGTCGGAAGTATAGTTTCT  ATGGCCGCGGGAGCT  GTTGAAATACAGTTCCTTCGTCAG  ATGGATAGACTGAAGCTGTCCA  ATGGATAGACTGAAGCTGTCCA  CCCCGATCCCTCAAATGCTATTGTGGAT  GCATTTGAGGGATCGGGGGACGGACAGGA  TCAAATGATATTGTG GCT GGCAAGTTC  GCCACAATATCATTTGAGGGATCGGGGG  CAACATCGAGGTGGCCAGTGATGTCTC  TGTAGCTCCACCGGTCACTACAGAGTA  GGAGGTTTCGAAGTCGCCAGCTCTGCTAAG  CCAAAGCTTCAGCGGTCGAGACGATTCTTT  TAATACGACTCACTATAGGG  TGCTAGTTATTGCTCAGCGG  TAAGAAGGAGATATACATATGATGTTTTCCAAGGCCACTGCC  GTGGTGGTGGTGGTGCTCGAG CTCCGGCCCGTTGCACAA  TAAGAAGGAGATATACATATG ATGTTTGCCACAGCGACCA  GTGGTGGTGGTGGTGCTCGAGAACACAGACGGTCAATGAGG  TAAGAAGGAGATATACATATGATGTCGGTGAATGGACCC  GTGGTGGTGGTGGTGCTCGAGAGAAATACATCTCTTTAGTCAG  GATATC ATGTCTAACATGGCGGAAGAC GATATC CTTTTTTGGTCGGAAGTATAGTTTCT  TAAGAAGGAGATATACATATGATGGCCGCGGGAGCT  GTGGTGGTGGTGGTGCTCGAGATGGCCGCGGGAGCT  GGAATTCCATATGATGCATGATGAGGCGGAGTACTAC  CCGGAATTC TACGAACTTGAGGACAAGCTTCT  CATGGTGGCGACCGGTGGATCATGTTTTCCAAGGCCACTGCC  TCAGATCTCGAGCTCAAGCTTCTCCGGCCCGTTGCACAA  CATGGTGGCGACCGGTGGATCATGTTTGCCACAGCGACCA  TCAGATCTCGAGCTCAAGCTTAACACAGACGGTCAATGAGG  CATGGTGGCGACCGGTGGATCATCCACAATATCATTTGAGGGATC  TCAGATCTCGAGCTCAAGCTTATGGGCAAGTTCAATGGGGAG  CATGGTGGCGACCGGTGGATCGTCCACCTCGATGTTGTTGC  TCAGATCTCGAGCTCAAGCTTATGAGTGATGTCTCATTGGAGATCC  CATGGTGGCGACCGGTGGATCGTCGACTTCGAAACCTCCGTT  TCAGATCTCGAGCTCAAGCTTATGTCTGCTAAGAAAGGACTGTTG  ATCCAAGCTTCTGCAGGAATTCATGTCGGTGAATGGACCC  TTTCTGCTCTCTAGACTCGAG AGAGAAATACATCTCTTTAGTCAG  ATCCAAGCTTCTGCAGGAATTCATGTCCAACACGGCGG  TTTCTGCTCTCTAGACTCGAGCTTTTTTGGTCGGAAGTATAGTTTCT  ATCCAAGCTTCTGCAGGAATTCATGGCCGCGGGAGCT  TTTCTGCTCTCTAGACTCGAG GTTGAAATACAGTTCCTTCGTCAG  TTTCACCCTCGGCAACTTGT  AGCCTTGGTGTCCAGCTTAC  CAATGTGTTGCAGCCACGAA  TGCGGTCCTTTCAAAGACGA  CGTGCGTGACATCAAGGAG  AGGAAGGAAGGCTGGAAGAG |
