# Supplementary figures and images for: GSDMEa-mediated pyroptosis is bi-directionally regulated by caspase and required for effective bacterial clearance in teleost
Source: Cell Death Dis. 2022 May 24;13(5):491. doi: 10.1038/s41419-022-04896-5 (PMC9130220; doi:10.1038/s41419-022-04896-5)

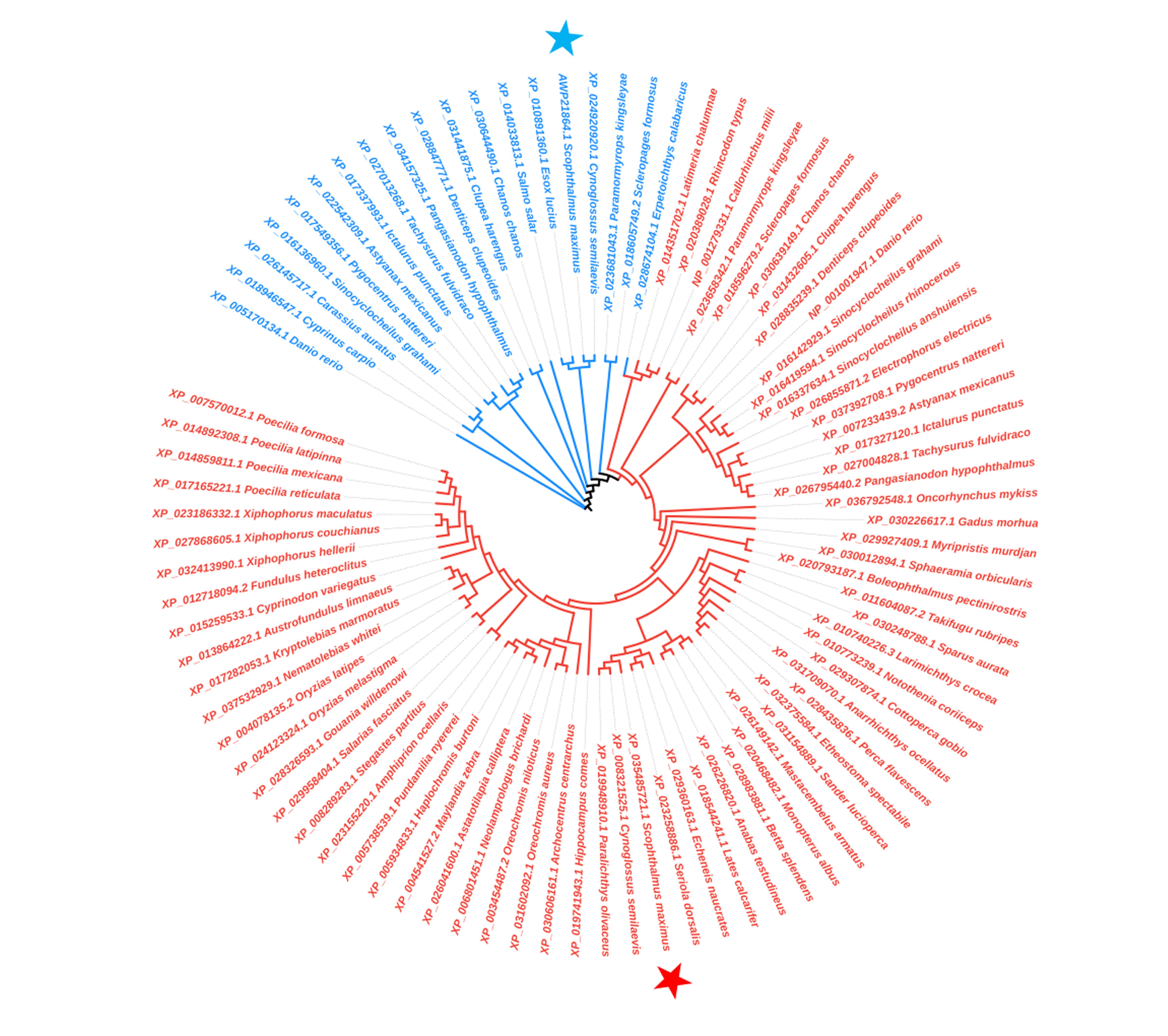

Supplement: Supplementary file 4 — Fig. S1 [file 41419_2022_4896_MOESM4_ESM.png]

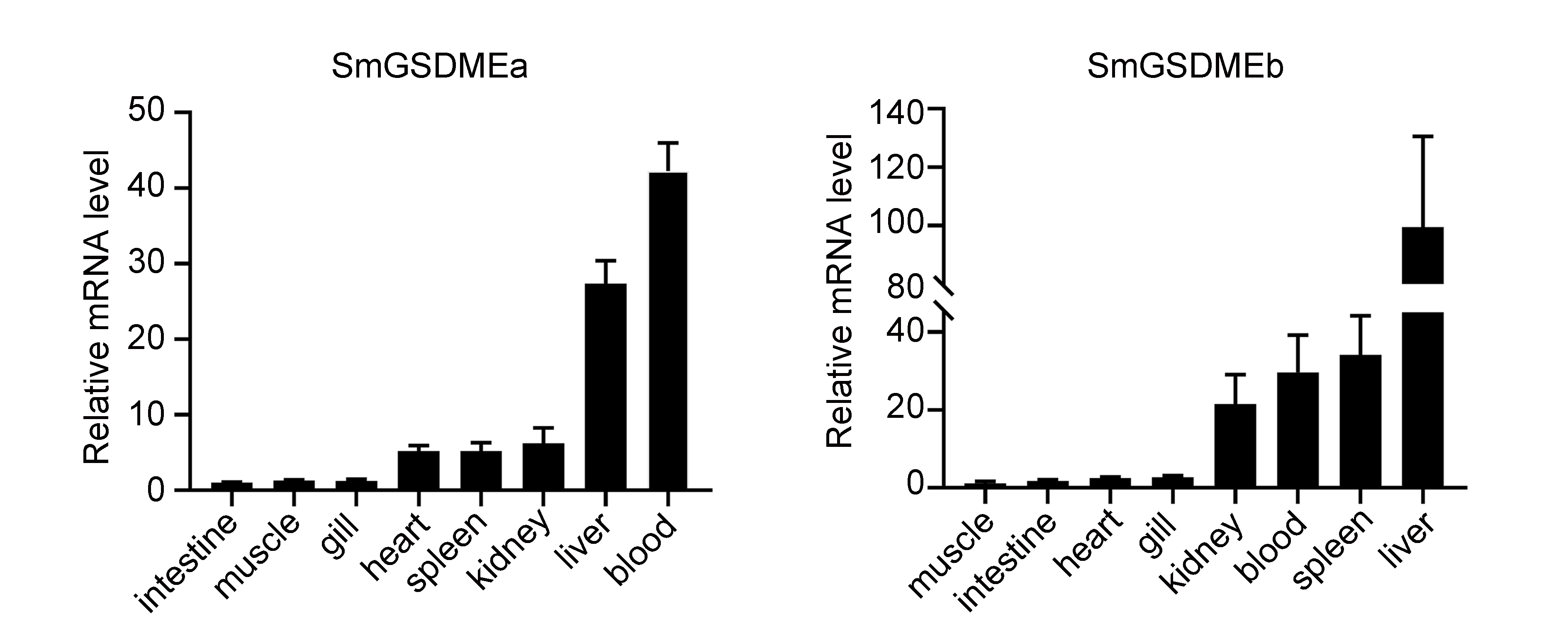

Supplement: Supplementary file 5 — Fig. S2 [file 41419_2022_4896_MOESM5_ESM.png]

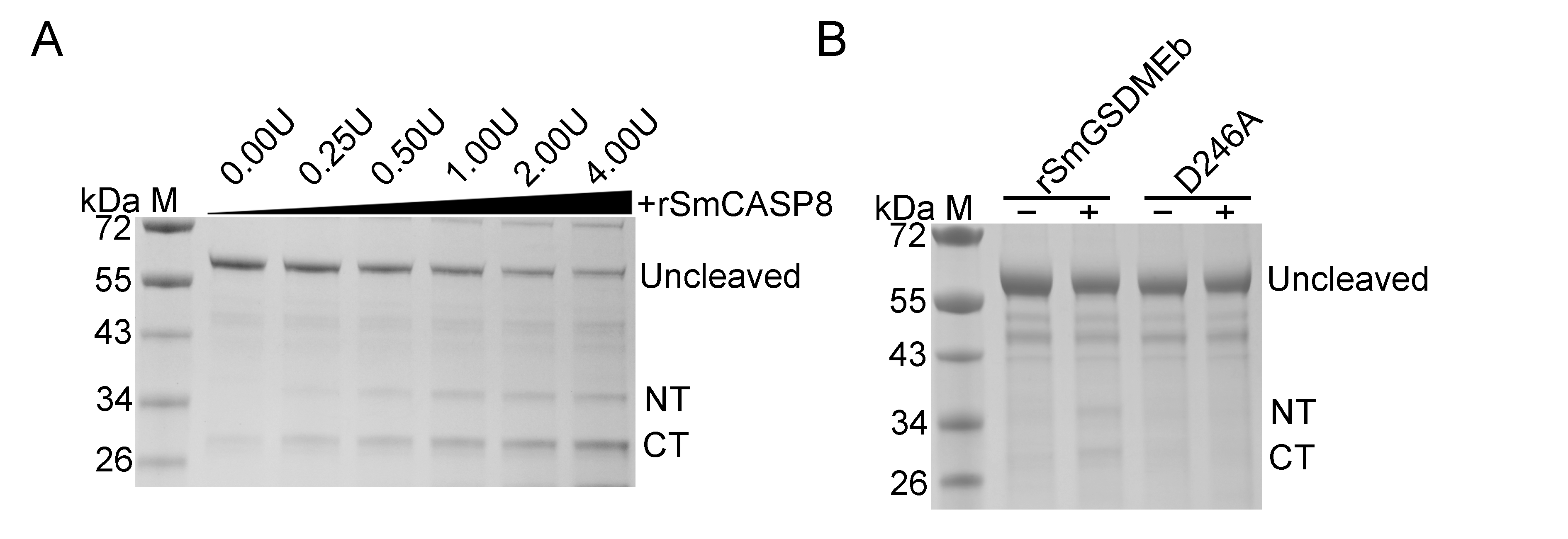

Supplement: Supplementary file 6 — Fig. S3 [file 41419_2022_4896_MOESM6_ESM.png]

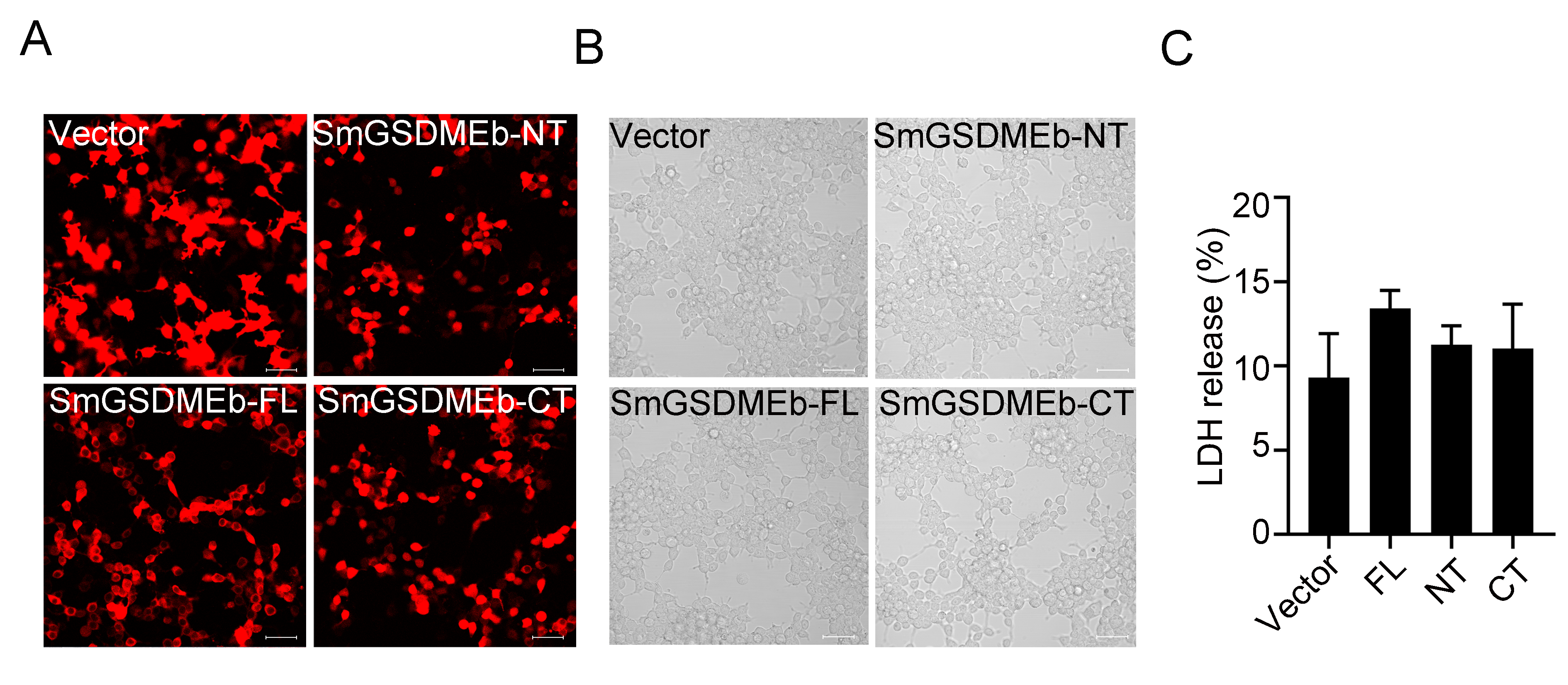

Supplement: Supplementary file 7 — Fig. S4 [file 41419_2022_4896_MOESM7_ESM.png]

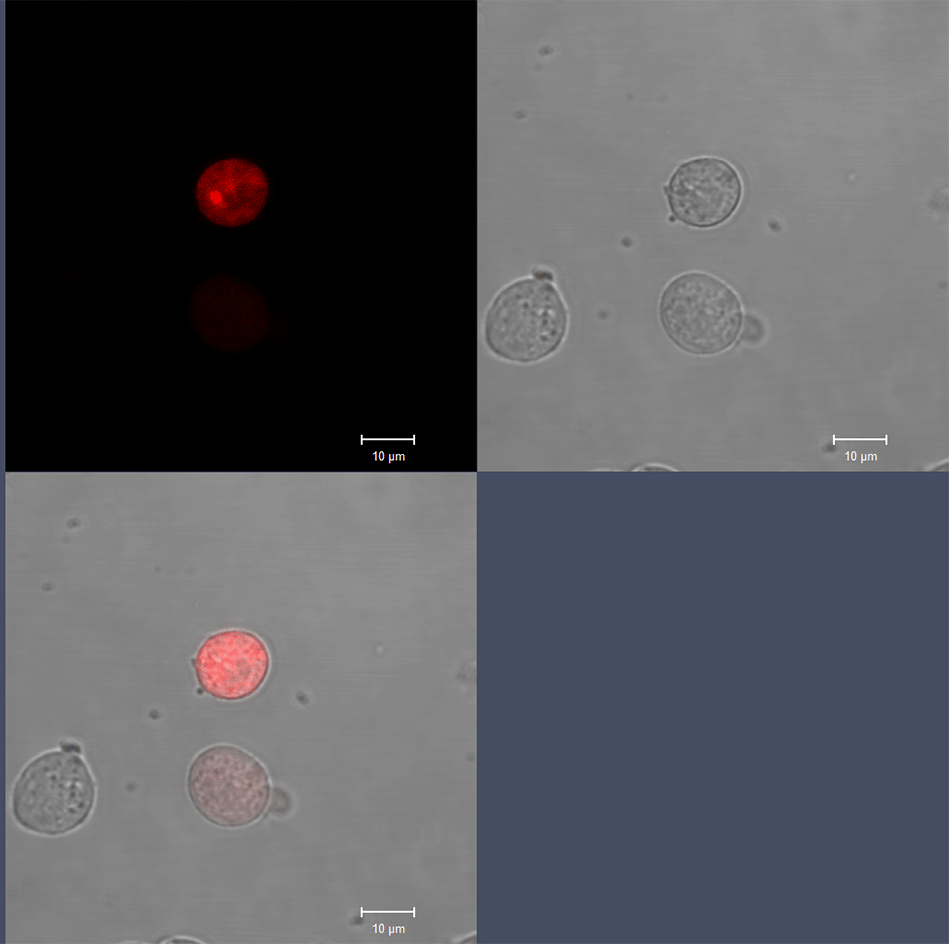

Supplement: Supplementary file 8 — Movie S1 [file 41419_2022_4896_MOESM8_ESM.gif]

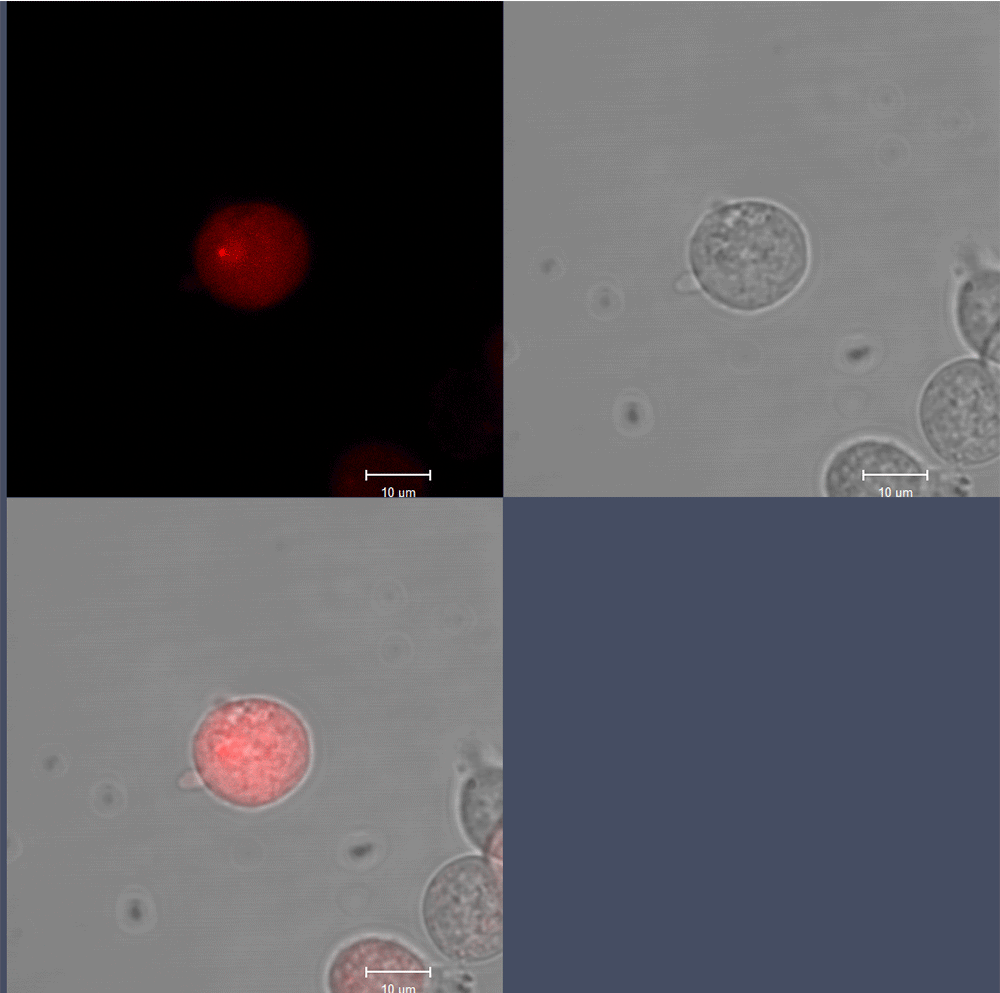

Supplement: Supplementary file 9 — Movie S2 [file 41419_2022_4896_MOESM9_ESM.gif]
